# Potent carbonic anhydrase I, II, IX and XII inhibition activity of novel primary benzenesulfonamides incorporating bis-ureido moieties

**DOI:** 10.1080/14756366.2023.2185762

**Published:** 2023-03-02

**Authors:** Tuba Tekeli, Suleyman Akocak, Andrea Petreni, Nebih Lolak, Servet Çete, Claudiu T. Supuran

**Affiliations:** a Vocational School of Technical Science, Department of Chemistry and Chemical Processing Technologies, Adıyaman University, Adıyaman, Türkiye; b Department of Chemistry, Faculty of Science, Gazi University, Ankara, Türkiye; c Department of Pharmaceutical Chemistry, Faculty of Pharmacy, Adıyaman University, Adıyaman, Türkiye; d NEUROFARBA Dept., Sezione di Scienze Farmaceutiche, Università degli Studi di Firenze, Sesto Fiorentino (Florence), Italy

**Keywords:** Bis-ureido, carbonic anhydrase, sulphonamide, isoform-selective inhibitor, anticancer agent

## Abstract

A novel series of twelve aromatic bis-ureido-substituted benzenesulfonamides was synthesised by conjugation of aromatic aminobenzenesulfonamides with aromatic bis-isocyanates. The obtained bis-ureido-substituted derivatives were tested against four selected human carbonic anhydrase isoforms (hCA I, hCA II, hCA IX and hCA XII). Most of the new compounds showed an effective inhibitory profile against isoforms hCA IX and hCA XII, also having some selectivity with respect to hCA I and hCA II. The inhibition constants of these compounds against isoforms hCA IX and XII were in the range of 6.73–835 and 5.02–429 nM, respectively. Since hCA IX and hCA XII are important drug targets for anti-cancer/anti-metastatic drugs, these effective inhibitors reported here may be considered of interest for cancer related studies in which these enzymes are involved.

## Introduction

Carbonic anhydrases (CAs, EC 4.2.1.1) are a superfamily of abundant metalloenzymes that catalyse a physiologically very important reaction, which is the reversible conversion of the carbon dioxide (CO_2_) to bicarbonate (HCO_3_^−^) and proton ions (H^+^). This reaction is also occurring under noncatalytic conditions but it is very slow for most life processes in which it is involved^1–8^. Up to now, eight genetically distinct CA families (α, β, γ, δ, ζ, η, θ, and more recently ι) were reported and they are present in all kingdoms of life^9–16^. Among them, in humans, 16 different isozymes which are all belong to the α-CAs were discovered, possessing a diverse sub-cellular localisation, catalytic activity and organ/tissue distribution^17–24^. These isoforms were classified according to their localisation, including the cytosolic forms (CA I, II, III, VII, and XIII), membrane-bound ones (CA IV, IX, XII, XIV, and XV), mitochondrial isoforms (CA VA and VB), and one secreted in saliva and milk (CA VI)^21–26^. Among these isoforms, two of the membrane-bound ones (CA IX and CA XII), have been identified as tumour-associated enzymes and attracted much attention in the search of novel, potent and selective cancer drugs, in the last decades^17–24^. These isoforms have a crucial role as diagnostic tools for imaging hypoxic tumours but also in the metabolism, survival, migration and invasion of tumour cells, by regulating pH and other processes connected to tumorigenesis. Therefore, potent and selective inhibitors of these isoforms with new class of compounds might be a strategy to develop efficient antitumor/antimetastatic agents^17–24^.

**Figure 1.**
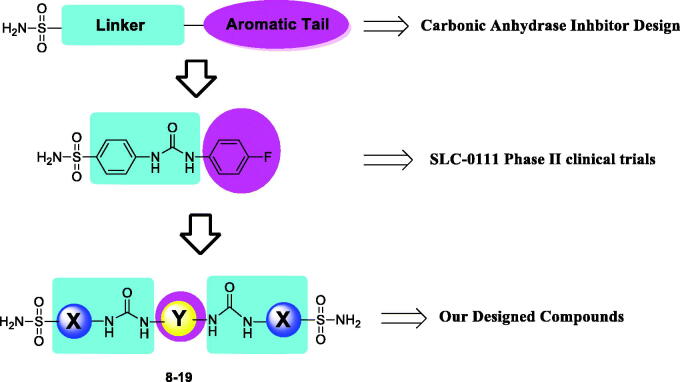
The design strategy of novel bis-ureido substituted primary benzenesulfonamides **8–19**.

Ureido-substituted primary benzenesulfonamides were extensively studied as potent and selective CA inhibitors (CAIs), and among them 4-[[(4-fluorophenyl) carbamoyl] amino] benzenesulfonamide (SLC-0111) (Figure 1) has reached to Phase Ib/II clinical trials for the management of advanced metastatic solid tumors^12–14^. Initially, ureido-substituted benzenesulfonamides were investigated as hCA I, hCA II, and bCA IV inhibitors possessing high activity and unexpectedly high selectivity^25^. Later, other such ureido bearing compounds (benzenesulfonamides and benzenesulfamates) have been designed, synthesised and explored as potent and selective hCA IX and XII inhibitors^12–14^. On the other hand, more recently, secondary and tertiary benzenesulfonamides started to be also investigated as CAIs, some of which showed strong affinity against several important isozymes, although they were generally less effective, compared to primary counterparts^26^^,^^27^.

In continuation of our recent efforts to develop effective and isoform selective CAIs^28–30^, in the present study, we report bis-ureido-substituted primary benzenesulfonamides acting as potent human (h) hCA inhibitors using the design strategy summarised in Figure 1. To the best of our knowledge, this is the first bis-ureido-substituted primary benzenesulfonamide study, which examined the inhibition profile of these compounds on selected hCAs, namely off-target cytosolic isoforms hCA I and II, and tumour-overexpressed membrane-bound isoforms hCA IX and XII.

## Materials and methods

### Chemistry

Unless otherwise noted, all the chemicals and anhydrous solvents were purchased from Sigma-Aldrich, Merck, Alfa Aesar and TCI and used without further purification. Melting points (mp) were determined with SMP20 melting point apparatus and are uncorrected. FT-IR spectra were obtained by using Perkin Elmer Spectrum 100 FT-IR spectrometer. Nuclear Magnetic Resonance (^1^H-NMR and ^13^C-NMR) spectra of compounds were recorded using a Bruker Advance III 300 Mhz spectrometer in DMSO-d_6_ as the solvent, and TMS as the internal standard operating at 300 Mhz for ^1^H-NMR and 75 Mhz for ^13^C-NMR. Chemical shifts are expressed in ppm relative to tetramethylsilane. Splitting patterns are designated as singlet (s), doublet (d), triplet (t), quartette (q), and multiplet (m). Thin layer chromatography (TLC) was carried out on Merck silica gel 60 F_254_ plates.

### General procedure for preparation of bis-ureido substituted primary benzenesulfonamide derivatives (8-19)

**Scheme 1.**
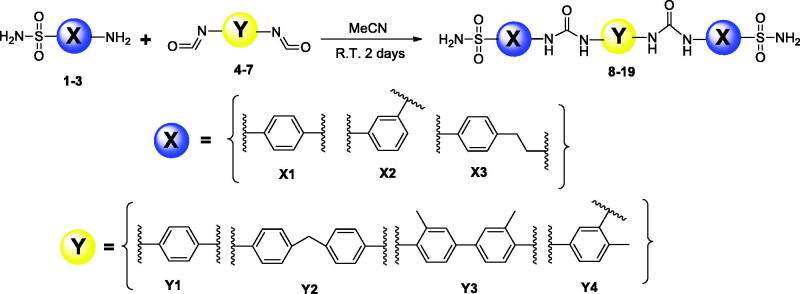
General synthetic route for the synthesis of the primary benzenesulfonamides incorporating bis-ureido moieties **8–19**.

4-aminobenzenesulfonamide/3-aminobenzenesulfonamide/4(2-aminoethyl)benzenesulfonamide (2 mmol) was dissolved in acetonitrile (10-15 ml) and then treated with subsequent bis-isocyanates (1.1 mmol) (1,4-Phenylene diisocyanide for **Y1**, 4,4′-Methylenebis (phenyl isocyanate) for **Y2**, 3,3′-Dimethyl-4,4′-biphenylene diisocyanate for **Y3,** 4-Methyl-1,3-phenylene diisocyanate for **Y4**). The mixture was stirred at room temperature for 3h, and then heated at 50 °C until completion (TLC monitoring). The obtained precipitate was filtered off, washed with diethyl ether (50 ml) and water, and dried *in vacuo*. The obtained products (compounds **8-19**) were characterised in detail by spectroscopic and analytic methods (FT-IR, ^1^H-NMR, ^13^C-NMR, and melting points) (Scheme 1).

#### 4,4'-(((1,4-Phenylenebis(azanediyl))bis(carbonyl))bis(azanediyl))dibenzenesulfonamide (8)


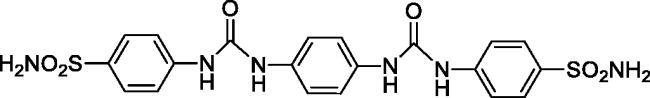


Yield: 88%; Colour: white solid; Melting Point: >300 °C; FT-IR (cm^−1^): 3387, 3331, 3222 (NH), 1689 (C=O), 1306, 1154 (symmetric) (S=O); ^1^H-NMR (DMSO-d_6_, 300 MHz, δ ppm): 9.02 (s, 2H, -NH-), 8.69 (s, 2H, -NH-), 7.73 (d, *J* = 7.5 Hz, 4H, Ar-H), 7.61 (d, *J* = 7.5 Hz, 4H, Ar-H), 7.40 (s, 4H, -SO_2_NH_2_), 7.21 (s, 4H, Ar-H); ^13^C-NMR (DMSO-d_6_, 75 MHz, δ ppm): 150.65, 141.31, 135.00, 132.30, 125.16, 117.57, 115.61.

#### 4,4'-((((Methylenebis(4,1-phenylene))bis(azanediyl))bis(carbonyl))bis(azanediyl))dibenzenesulfonamide (9)


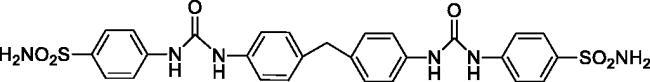


Yield: 82%; Colour: white solid; Melting Point: 293–294 °C; FT-IR (cm^−1^): 3374, 3324, 3262 (NH), 1656 (C=O), 1328, 1151 (symmetric) (S=O); ^1^H-NMR (DMSO-d_6_, 300 MHz, δ ppm): 9.05 (s, 2H, -NH-), 8.76 (s, 2H, -NH-), 7.75 (d, *J* = 8.4 Hz, 4H, Ar-H), 7.63 (d, *J* = 8.7 Hz, 4H, Ar-H), 7.40 (d, *J* = 8.4 Hz, 4H, Ar-H), 7.25 (s, 4H, -SO_2_NH_2_), 7.15 (d, *J* = 8.7 Hz, 4H, Ar-H), 3.84 (s, 2H, Ph-CH_2_-Ph); ^13^C-NMR (DMSO-d_6_, 75 MHz, δ ppm): 152.74, 143.39, 137.64, 137.18, 135.87, 129.46, 127.31, 119.11, 118.82, 117.85.

#### 4,4'-((((3,3'-Dimethyl-[1,1'-biphenyl]-4,4'-diyl)bis(azanediyl))bis(carbonyl))bis(azanediyl))dibenzenesulfonamide (10)


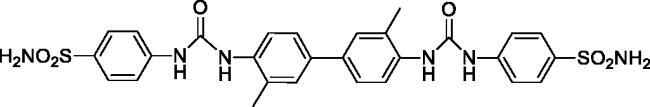


Yield: 76%; Colour: white solid; Melting Point: >300 °C; FT-IR (cm^−1^): 3412, 3348, 3292 (NH), 1693 (C=O), 1304, 1148 (symmetric) (S=O); ^1^H-NMR (DMSO-d_6_, 300 MHz, δ ppm): 9.45 (s, 2H, -NH-), 8.13 (s, 2H, -NH-), 7.99–7.95 (m, 4H, Ar-H), 7.77 (d, *J* = 8.4 Hz, 4H, Ar-H), 7.66 (d, *J* = 8.4 Hz, 4H, Ar-H), 7.52–7.45 (m, 4H, Ar-H), 7.25 (s, 4H, -SO_2_NH_2_), 2.30 (s, 6H, -CH_3_); ^13^C-NMR (DMSO-d_6_, 75 MHz, δ ppm): 152.76, 146.07, 143.43, 137.11, 136.16, 134.18, 128.56, 127.60, 124.20, 121.99, 117.31, 18.61.

#### 4,4'-((((4-Methyl-1,3-phenylene)bis(azanediyl))bis(carbonyl))bis(azanediyl))dibenzenesulfonamide (11)


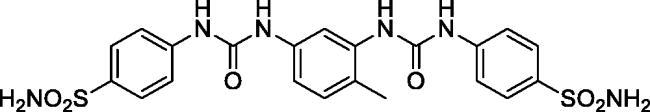


Yield: 63%; Colour: white solid; Melting Point: 279–281 °C; FT-IR (cm^−1^): 3391, 3309, 3190 (NH), 1701 (C=O), 1310, 1154 (symmetric) (S=O); ^1^H-NMR (DMSO-d_6_, 300 MHz, δ ppm): 9.45 (s, 1H, -NH-), 8.94 (s, 1H, -NH-), 8.80 (d, *J* = 7.5 Hz, 2H, Ar-H), 8.44 (s, 1H, -NH-), 8.29 (s, 1H, -NH-), 8.04 (s, 2H, Ar-H), 7.78–7.59 (m, 5H, Ar-H), 7.23 (s, 4H, -SO_2_NH_2_), 7.18–7.08 (m, 2H, Ar-H), 2.21 (s, 3H, -CH_3_); ^13^C-NMR (DMSO-d_6_, 75 MHz, δ ppm): 152.74, 152.38, 147.11, 143.42, 138.20, 137.83, 137.40, 130.61, 127.21, 121.89, 117.63, 115.96, 111.67, 104.71, 17.63.

#### 3,3'-(((1,4-Phenylenebis(azanediyl))bis(carbonyl))bis(azanediyl))dibenzenesulfonamide (12)


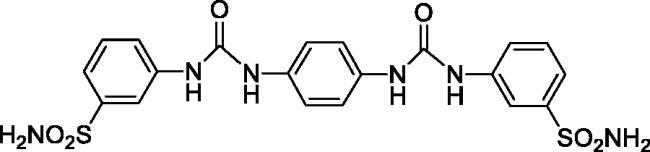


Yield: 92%; Colour: white solid; Melting Point: >300 °C; FT-IR (cm^−1^): 3362, 3322, 3267 (NH), 1638 (C=O), 1338, 1153 (symmetric) (S=O); ^1^H-NMR (DMSO-d_6_, 300 MHz, δ ppm): 8.96 (s, 2H, -NH-), 8.61 (s, 2H, -NH-), 8.07 (s, 2H, Ar-H), 7.55 (d, *J* = 7.2 Hz, 2H, Ar-H), 7.46 (t, 2H, Ar-H), 7.42–7.40 (m, 6H, Ar-H and -SO_2_NH_2_), 7.36 (s, 4H, Ar-H); ^13^C-NMR (DMSO-d_6_, 75 MHz, δ ppm): 152.96, 145.43, 140.78, 134.47, 129.87, 121.43, 119.67, 119.17, 115.44.

#### 3,3'-((((Methylenebis(4,1-phenylene))bis(azanediyl))bis(carbonyl))bis(azanediyl))dibenzenesulfonamide (13)


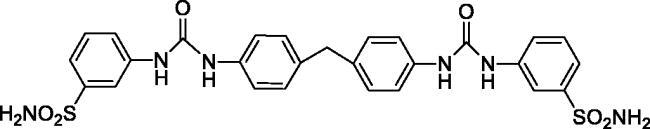


Yield: 82%; Colour: white solid; Melting Point: 293–295 °C; FT-IR (cm^−1^): 3357, 3322, 3268 (NH), 1644 (C=O), 1339, 1154 (symmetric) (S=O); ^1^H-NMR (DMSO-d_6_, 300 MHz, δ ppm): 8.99 (s, 2H, -NH-), 8.68 (s, 2H, -NH-), 8.09 (s, 2H, Ar-H), 7.54 (d, *J* = 7.5 Hz, 2H, Ar-H), 7.50–7.41 (m, 8H, Ar-H), 7.38 (s, 4H, -SO_2_NH_2_), 7.15 (d, *J* = 8.4 Hz, 4H, Ar-H), 3.84 (s, 2H, Ph-CH_2_-Ph); ^13^C-NMR (DMSO-d_6_, 75 MHz, δ ppm): 152.88, 145.14, 140.71, 137.78, 135.80, 129.88, 129.44, 121.46, 119.23, 119.04, 115.43.

#### 3,3'-((((3,3'-Dimethyl-[1,1'-biphenyl]-4,4'-diyl)bis(azanediyl))bis(carbonyl))bis(azanediyl))dibenzenesulfonamide (14)


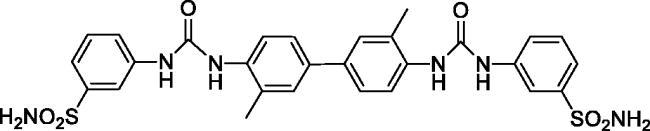


Yield: 80%; Colour: white solid; Melting Point: >300 °C; FT-IR (cm^−1^): 3367, 3306, 3284 (NH), 1639 (C=O), 1302, 1151 (symmetric) (S=O); ^1^H-NMR (DMSO-d_6_, 300 MHz, δ ppm): 9.42 (s, 2H, -NH-), 8.13 (s, 2H, -NH-), 8.06 (s, 2H, Ar-H), 7.96 (d, *J* = 8.4 Hz, 2H, Ar-H), 7.60 (d, *J* = 7.5 Hz, 2H, Ar-H), 7.52–7.46 (m, 8H, Ar-H), 7.40 (s, 4H, -SO_2_NH_2_), 2.34 (s, 6H, -CH_3_); ^13^C-NMR (DMSO-d_6_, 75 MHz, δ ppm): 153.04, 145.20, 140.78, 136.71, 134.75, 130.01, 128.46, 124.47, 121.73, 121.28, 119.24, 115.27, 18.51.

#### 3,3'-((((4-Methyl-1,3-phenylene)bis(azanediyl))bis(carbonyl))bis(azanediyl))dibenzenesulfonamide (15)


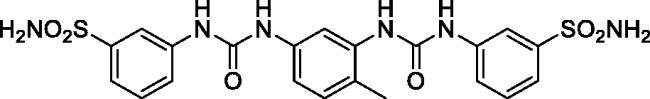


Yield: 76%; Colour: white solid; Melting Point: 233–234 °C; FT-IR (cm^−1^): 3388, 3322, 3272 (NH), 1692, 1645 (C=O), 1308, 1150 (symmetric) (S=O); ^1^H-NMR (DMSO-d_6_, 300 MHz, δ ppm): 9.42 (s, 1H, -NH-), 8.88 (s, 1H, -NH-), 8.77 (s, 1H, -NH-), 8.17 (s, 1H, Ar-H), 8.10 (d, *J* = 7.8 Hz, 2H, Ar-H), 7.98 (s, 1H, Ar-H), 7.56–7.45 (m, 6H, Ar-H), 7.39 (s, 5H, -SO_2_NH_2_ and Ar-H), 7.18 (d, *J* = 7.2 Hz, 1H, Ar-H), 7.10 (d, *J* = 8.4 Hz, 1H, Ar-H), 2.21 (s, 3H, -CH_3_); ^13^C-NMR (DMSO-d_6_, 75 MHz, δ ppm): 152.87, 152.78, 145.19, 140.76, 138.07, 137.83, 130.79, 130.03, 129.89, 121.44, 121.31, 121.21, 119.21, 115.33, 113.53, 111.38, 17.71.

#### 4,4'-((((1,4-Phenylenebis(azanediyl))bis(carbonyl))bis(azanediyl))bis(ethane-2,1-diyl))dibenzenesulfonamide (16)


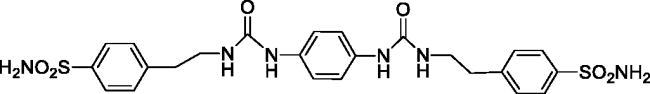


Yield: 88%; Colour: white solid; Melting Point: >300 °C; FT-IR (cm^−1^): 3361, 3333, 3266 (NH), 1626 (C=O), 1333, 1156 (symmetric) (S=O); ^1^H-NMR (DMSO-d_6_, 300 MHz, δ ppm): 8.28 (s, 2H, -CH_2_CH_2_NH-CO-NH-), 7.77 (d, *J* = 8.4 Hz, 4H, Ar-H), 7.43 (d, *J* = 8.4 Hz, 4H, Ar-H), 7.33 (s, 4H, -SO_2_NH_2_), 7.23 (s, 4H, Ar-H), 6.05 (t, 2H, -CH_2_CH_2_NH-CO-NH-), 3.35 (q, 4H, -CH_2_CH_2_NH-CO-NH-), 2.82 (t, 4H, -CH_2_CH_2_NH-CO-NH-); ^13^C-NMR (DMSO-d_6_, 75 MHz, δ ppm): 155.76, 144.37, 142.47, 134.73, 129.65, 126.19, 118.88, 40.76, 36.11.

#### 4,4'-(((((Methylenebis(4,1-phenylene))bis(azanediyl))bis(carbonyl))bis(azanediyl))bis(ethane-2,1-diyl))dibenzenesulfonamide (17)


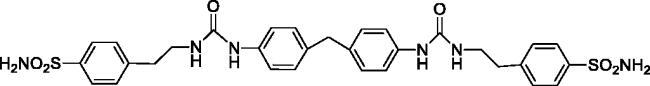


Yield: 86%; Colour: white solid; Melting Point: 270–272 °C; FT-IR (cm^−1^): 3361, 3335, 3268 (NH), 1636 (C=O), 1341, 1161 (symmetric) (S=O); ^1^H-NMR (DMSO-d_6_, 300 MHz, δ ppm): 8.40 (s, 2H, -CH_2_CH_2_NH-CO-NH-), 7.78 (d, *J* = 8.4 Hz, 4H, Ar-H), 7.42 (d, *J* = 8.4 Hz, 4H, Ar-H), 7.33 (s, 4H, -SO_2_NH_2_), 7.28 (d, *J* = 8.7 Hz, 4H, Ar-H), 7.04 (d, *J* = 8.4 Hz, 4H, Ar-H), 6.10 (t, 2H, -CH_2_CH_2_NH-CO-NH-), 3.75 (s, 2H, Ph-CH_2_-Ph), 3.35 (q, 4H, -CH_2_CH_2_NH-CO-NH-), 2.83 (t, 4H, -CH_2_CH_2_NH-CO-NH-); ^13^C-NMR (DMSO-d_6_, 75 MHz, δ ppm): 155.63, 144.53, 142.53, 138.79, 134.76, 129.64, 129.23, 126.19, 118.24, 40.70, 36.04.

#### 4,4'-(((((3,3'-Dimethyl-[1,1'-biphenyl]-4,4'-diyl)bis(azanediyl))bis(carbonyl))bis(azanediyl))bis(ethane-2,1-diyl))dibenzenesulfonamide (18)


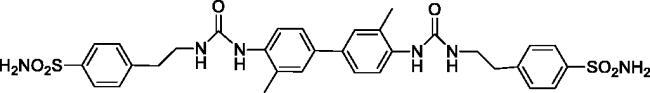


Yield: 85%; Colour: white solid; Melting Point: >300 °C; FT-IR (cm^−1^): 3365, 3330, 3276 (NH), 1629 (C=O), 1334, 1151 (symmetric) (S=O); ^1^H-NMR (DMSO-d_6_, 300 MHz, δ ppm): 7.89 (d, *J* = 8.4 Hz, 2H, Ar-H), 7.80 (d, *J* = 8.1 Hz, 4H, Ar-H), 7.73 (s, 2H, -CH_2_CH_2_NH-CO-NH-), 7.46 (d, *J* = 8.4 Hz, 4H, Ar-H), 7.40–7.35 (m, 8H, Ar-H and -SO_2_NH_2_), 6.62 (t, 2H, -CH_2_CH_2_NH-CO-NH-), 3.40 (q, 4H, -CH_2_CH_2_NH-CO-NH-), 2.86 (t, 4H, -CH_2_CH_2_NH-CO-NH-), 2.24 (s, 6H, -CH_3_); ^13^C-NMR (DMSO-d_6_, 75 MHz, δ ppm): 155.82, 144.40, 142.51, 137.55, 133.95, 129.69, 128.17, 127.55, 126.21, 124.22, 121.27, 40.75, 36.08, 18.57.

#### 4,4'-(((((4-Methyl-1,3-phenylene)bis(azanediyl))bis(carbonyl))bis(azanediyl))bis(ethane-2,1-diyl))dibenzenesulfonamide (19)


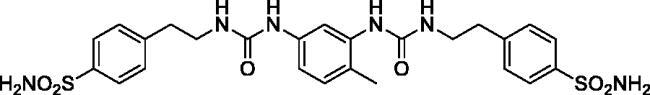


Yield: 79%; Colour: white solid; Melting Point: 245–247 °C; FT-IR (cm^−1^): 3344, 3309, 3261 (NH), 1627 (C=O), 1330, 1152 (symmetric) (S=O); ^1^H-NMR (DMSO-d_6_, 300 MHz, δ ppm): 8.41 (s, 1H, -CH_2_CH_2_NH-CO-NH-), 7.78 (dd, *J_1_* = 8.4 Hz, *J_2_* = 1.5 Hz, 5H, Ar-H), 7.59 (s, 1H, Ar-H), 7.43 (dd, *J_1_* = 8.4 Hz, *J_2_* = 2.4 Hz, 4H, Ar-H), 7.34 (s, 4H, -SO_2_NH_2_), 7.13 (d, *J* = 8.7 Hz, 1H, Ar-H), 6.95 (d, *J* = 8.7 Hz, 1H, Ar-H), 6.59 (t, 1H, -CH_2_CH_2_NH-CO-NH-), 6.02 (t, 1H, -CH_2_CH_2_NH-CO-NH-), 3.38(q, 4H, -CH_2_CH_2_NH-CO-NH-), 2.85 (t, 4H, -CH_2_CH_2_NH-CO-NH-), 2.01 (s, 3H, -CH_3_); ^13^C-NMR (DMSO-d_6_, 75 MHz, δ ppm): 155.75, 155.64, 144.37, 142.45, 138.95, 138.55, 130.44, 129.67, 126.21, 119.83, 112.10, 110.70, 40.73, 36.10, 17.70.

### CA inhibition

An SX.18 MV-R Applied Photophysics (Oxford, UK) stopped-flow instrument has been used to assay the catalytic/inhibition of various CA isozymes^31^. Phenol Red (at a concentration of 0.2 mM) has been used as an indicator, working at the absorbance maximum of 557 nm, with 10 mM Hepes (pH 7.4) as a buffer, 0.1 M Na_2_SO_4_ or NaClO_4_ (for maintaining constant the ionic strength; these anions are not inhibitory in the used concentration), following the CA-catalyzed CO_2_ hydration reaction for a period of 5–10 s. Saturated CO_2_ solutions in water at 25 °C were used as substrate. Stock solutions of inhibitors were prepared at a concentration of 10 mM (in DMSO-water 1:1, v/v) and dilutions up to 0.01 nM done with the assay buffer mentioned above. At least 7 different inhibitor concentrations have been used for measuring the inhibition constant. Inhibitor and enzyme solutions were pre-incubated together for 10 min at room temperature prior to assay, in order to allow for the formation of the E-I complex. Triplicate experiments were done for each inhibitor concentration, and the values reported throughout the paper is the mean of such results. The inhibition constants were obtained by nonlinear least-squares methods using the Cheng-Prusoff equation, as reported earlier^32–37^, and represent the mean from at least three different determinations^38–45^. All CA isozymes used here were recombinant proteins obtained as reported earlier by our group and their concentrations were in the range of 6–14 nM^38^–45.

## Results and discussion

### General synthesis and design strategy of the compounds

In recent studies, CAIs having bifunctional pharmacophores in their structures were reported, in order to improve biological activity and selectivity of compounds by ditopic interactions on the active site of the same or different enzyme(s)^46–50^. In the context of the bis-substituted CAI design approach, we focussed on the development of a new bis-ureido-substituted benzenesulfonamides. Our aim was to produce molecules which have two binding groups in their structure and the ureido linker in between them to investigate isoform selectivity and potency of compounds by a potentially synergistic/multivalent effect. On the other hand, we aimed to find the relationship between the effect of the zinc binding group (primary sulphonamide part) and the linker position and length (ureido part) against various CA isozyme of pharmacologic relevance. To achieve this, we have used three well known primary benzenesulfonamide pharmacophores namely, sulphanilamide, metanilamide and 4-(2-aminoethyl)benzenesulfonamide which have been converted to aromatic bis-ureido derivatives (**8-19**). Hence, these primary benzenesulfonamide derivatives were condensed with four different aromatic bis-isocyanate moieties under mild conditions to produce twelve novel bis-ureido-substituted benzenesulfonamides derivatives. The general synthetic route is shown in the Scheme 1. Briefly, acetonitrile was used as a solvent and the reaction temperature was from room temperature to 50°C, overnight.

### Carbonic anhydrase inhibition

All the newly synthesised bis-ureido-substituted primary benzenesulfonamide derivatives were evaluated for their CA inhibition properties against two cytosolic off-target isoforms (hCA I and II) and two membrane-bound isoforms (hCA IX and XII) by using a stopped-flow assay.The well-known CAI drug acetazolamide (AAZ) was used as a standard for comparison, and all the obtained results are summarised in Table 1. The following structure-activity relationship (SAR) can be drawn from obtained carbonic anhydrase inhibition studies as shown in the Table 1.

**Table 1.** Inhibition data of human CA isoforms hCA I, hCA II, hCA IX and hCA XII with bis-ureido-substituted primary benzenesulfonamide derivatives **(8–19)** reported here and standard sulphonamide inhibitor Acetazolamide (AAZ) by a stopped flow CO_2_ hydrase assay.

			K_I_^a^ (nM)			
Cmp	X	Y	hCA I	hCA II	hCA IX	hCA XII
**8**	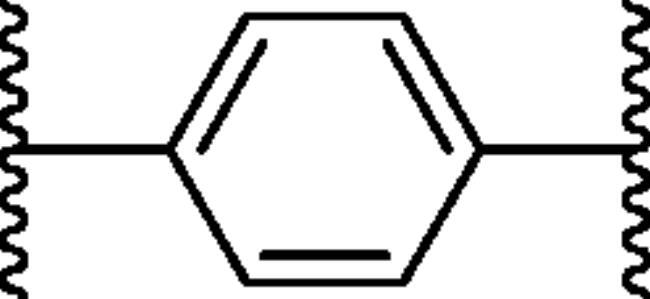	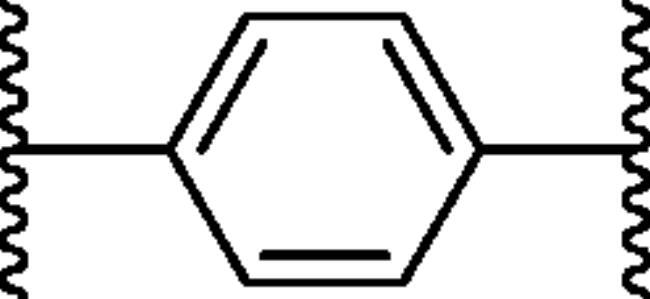	7279	680	57.5	37.3
**9**	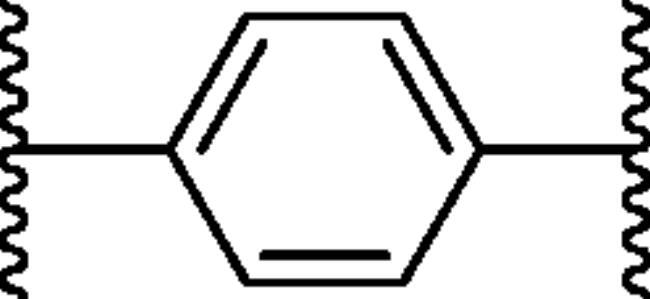	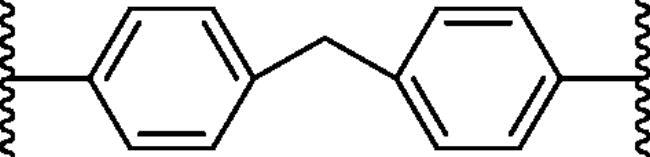	6282	484	835	429
**10**	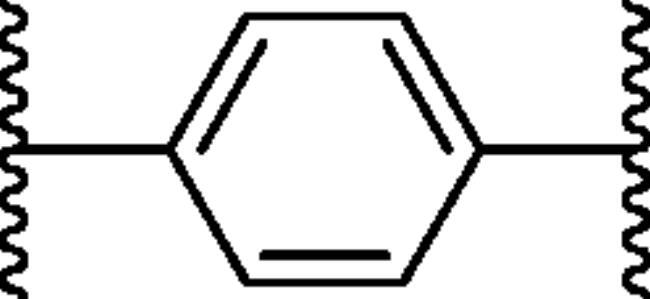	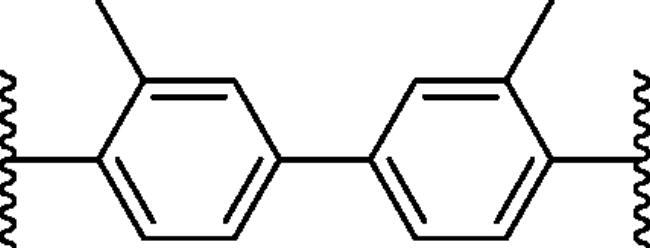	947	302	86.4	52.3
**11**	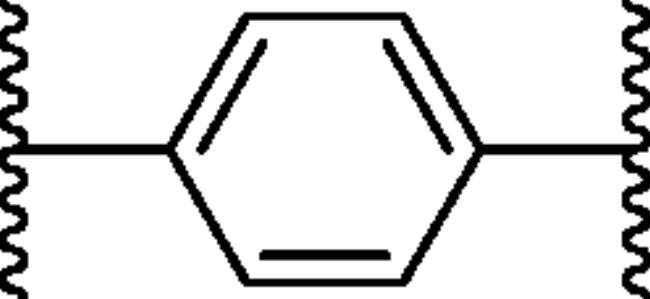	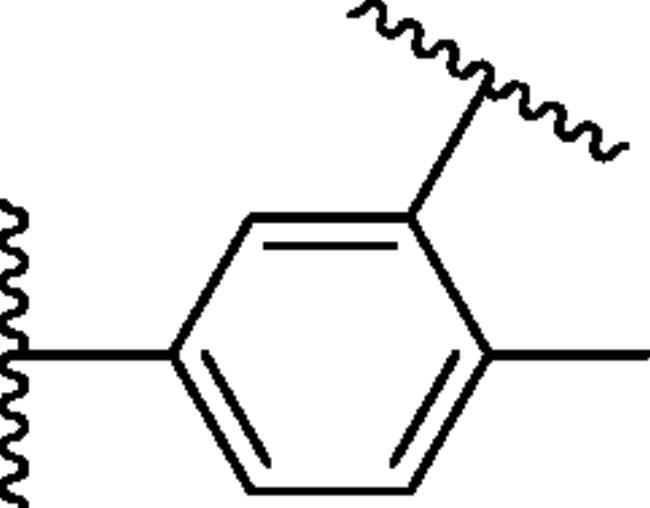	68.1	4.4	6.73	7.39
**12**	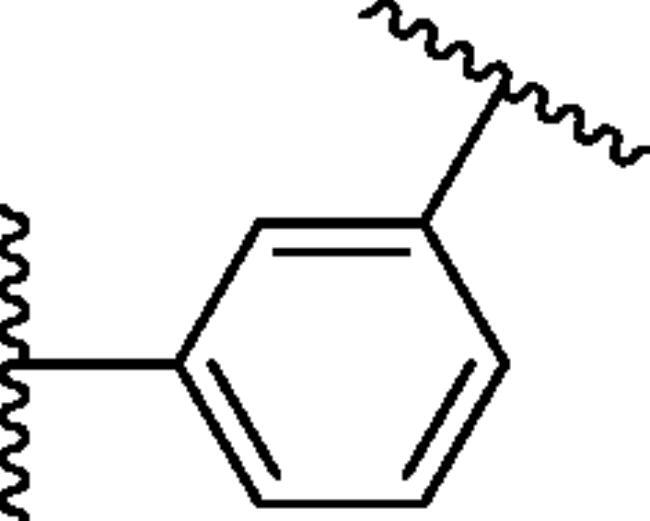	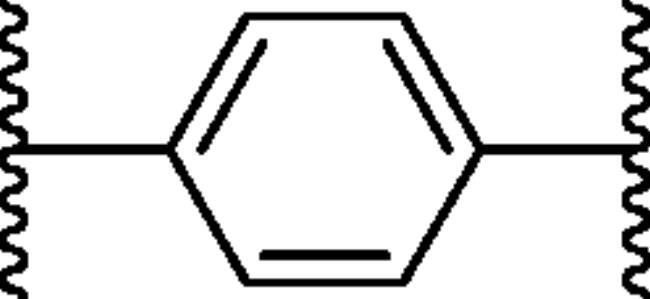	6519	550	63.8	63.2
**13**	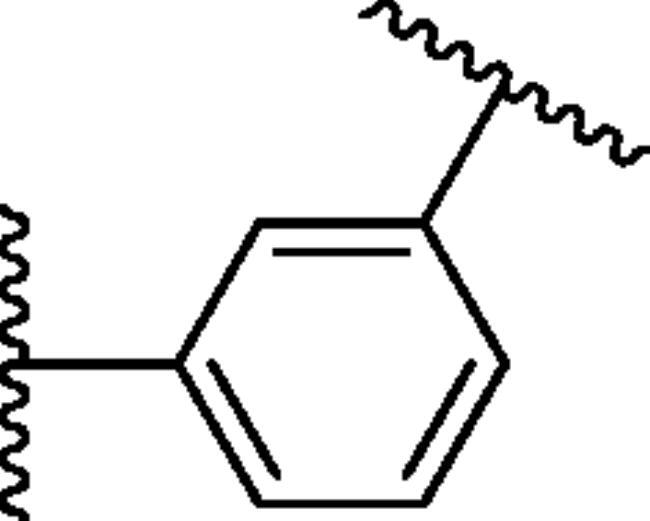	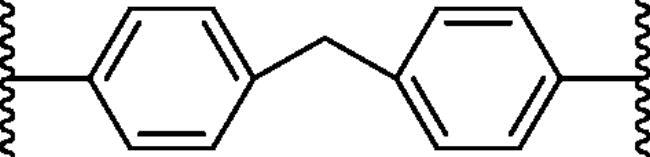	9174	306	78.7	27.7
**14**	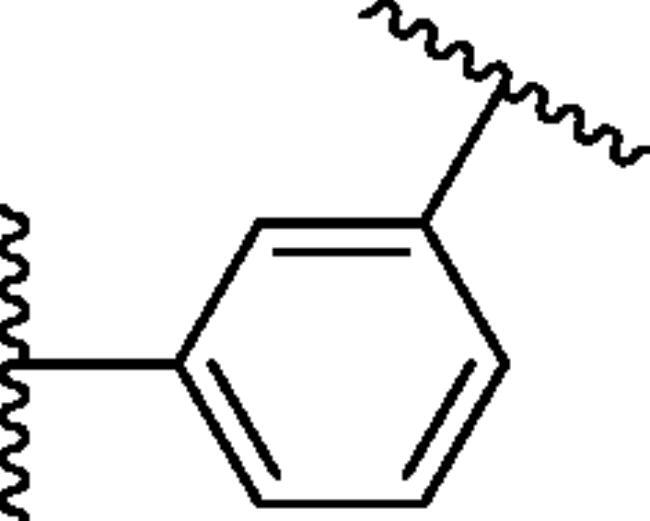	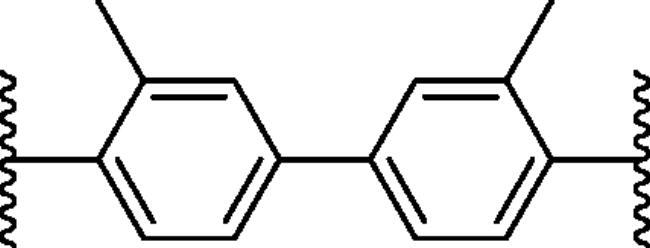	688	637	578	67.9
**15**	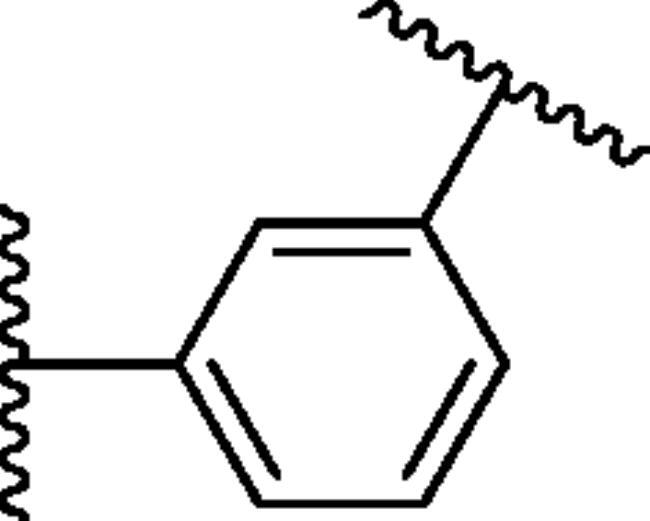	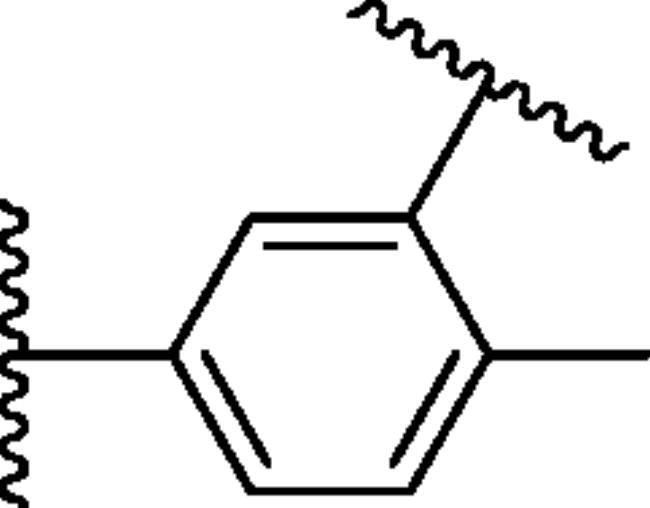	5002	80.8	81.1	67.1
**16**	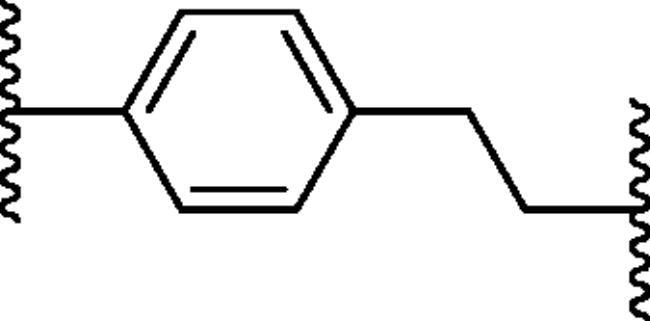	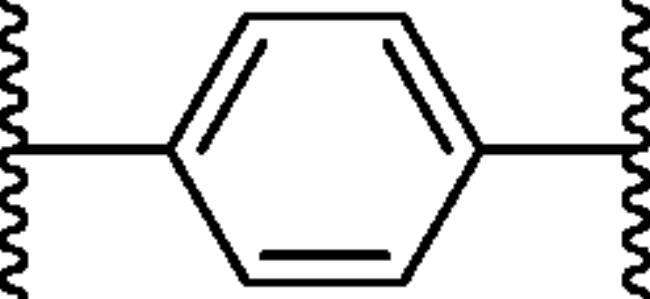	2915	424	68.7	82.8
**17**	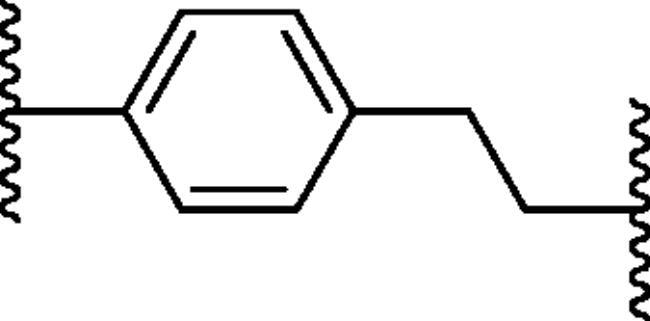	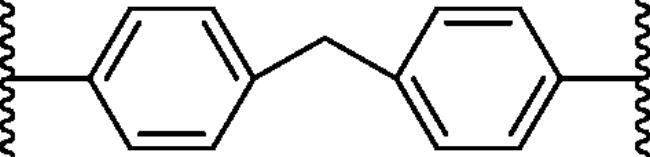	5351	792	91.9	33.6
**18**	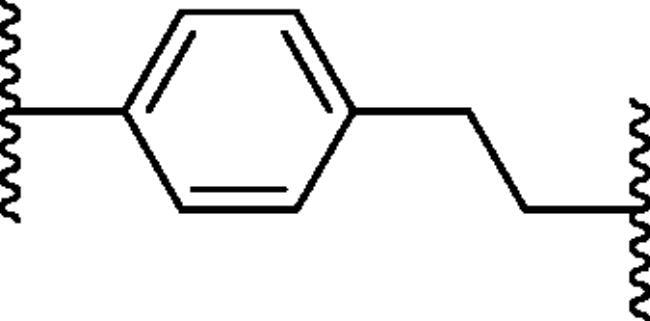	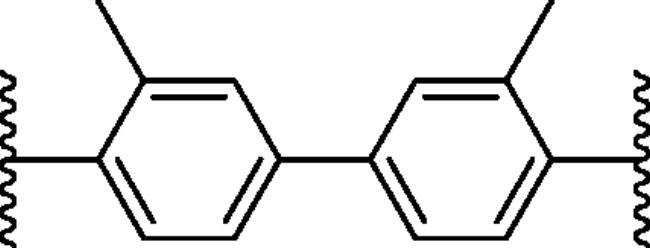	8313	604	93.7	60.3
**19**	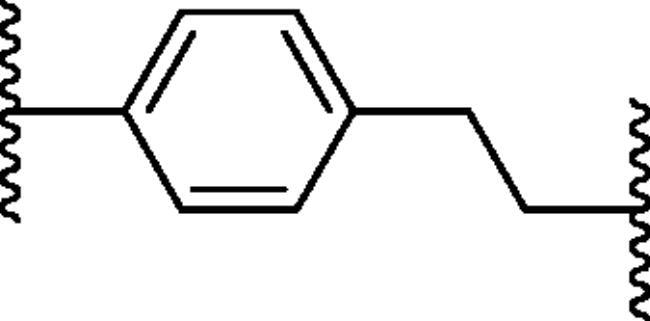	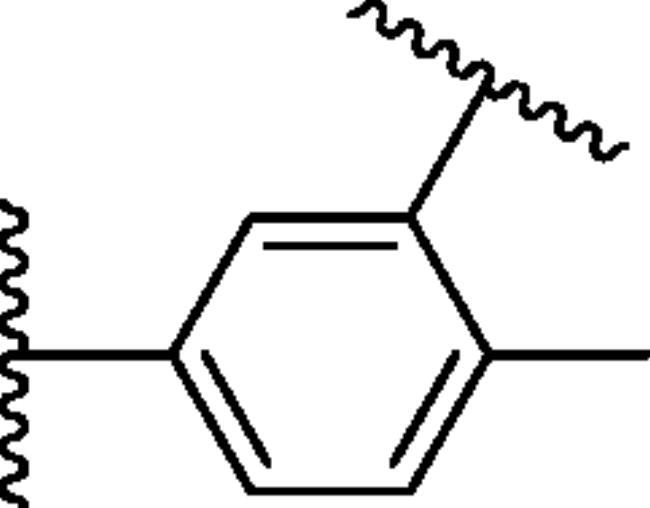	95.4	25.8	8.92	5.0
**AAZ**	–	–	250	12	2.5	25

^a^Errors in the range of ± standart error, from 3 different assays, by a stopped flow technique.

- In general, our designed compounds were less prone to inhibit the cytosolic off-target isoform hCA I, having K_i_ values of 68.1 to 9174 nM. The most potent compound was **11**, which is structurally different from other linkers by having substitution on 1,3- position in the phenyl ring although other ones have 1,4-substitution. Compound **19** showed good inhibition which is also have same linker with compound **11** by having K_i_ value of 95.4 nM. The other off-target isoform hCA II was effectively to moderately inhibited by the synthesised bis-ureido-substituted primary benzenesulfonamides. Interestingly, the most potent compound against hCA II was also compound **11** and **19**, as in hCA I inhibition, with a K_i_ values of 4.4 and 25.8 nM, respectively. Compound **11** showed the best inhibition value that observed in the present study against hCA II with K_i_ value of 4.4 nM.
- The tumour-overexpressed isoforms hCA IX and XII were efficiently inhibited, in general, by our novel compounds. More specifically, compound **11** showed a great affinity against these isoforms with K_i_ values of 6.73 and 7.39 nM, respectively. On the other hand, another compound from the same linker series compound **19**, was also shown great inhibition potency with K_i_ values of 8.92 and 5.02 nM, respectively. However, a better selectivity was observed for compound **19** against off target isoforms hCA I and hCA II. The remaining compounds in the present series also displayed great inhibition properties with K_i_ values ranging from 57.5 to 835 nM for hCA IX, and 27.7 to 429 nM for hCA XII.
- Overall, the SAR results demonstrate that 4-aminobenzenesulfonamide scaffold is a better primary sulphonamide as compared to other counterparts in combination with the bis-isocyanate (**Y4**) which has 1,3-substitution among others.

## Conclusions

A series of novel primary benzenesulfonamides incorporating bis-ureido moieties were synthesised through the conjugation of sulphonamides with aromatic bis-isocyanates under mild conditions using SLC-0111 as lead compound. These bifunctional CAI design strategy was applied to obtain potent and possibly selective inhibitors incorporating the ureido linker. The inhibition properties of the obtained compounds were evaluated against four selected CA isoforms, including cytosolic ones (hCA I and II), as well as the tumour-associated membrane-bound isoforms (hCA IX and XII). In general, these compounds showed weak hCA I inhibition with K_i_s ranging from 68.1 to 9174 nM and effective to moderate inhibition against another cytosolic isoform hCA II with K_i_s of 4.4 to 792 nM. The tumour-associated membrane-bound isoforms hCA IX and XII were effectively inhibited by some of these compounds with K_i_s in the range of 6.73–835 and 5.02–429 nM, respectively. Interestingly, compounds **11** and **19** were found to be potent derivatives against these isoforms in which both of them have the same ureido linker (1,3-substituted phenyl ring) in their structure. However, compound **19** was more selective against the off-target isoforms hCA I and II. As a result, the potent inhibition properties of these novel compounds against tumour-associated isoforms hCA IX and XII making these bis-ureido substituted compounds of interest for antimetastatic drug design research.

## Supplementary Material

Supplemental Material
