# Supplementary material for: Potent carbonic anhydrase I, II, IX and XII inhibition activity of novel primary benzenesulfonamides incorporating bis-ureido moieties
Source: J Enzyme Inhib Med Chem. 2023 Mar 2;38(1):2185762. doi: 10.1080/14756366.2023.2185762 (PMC9987750; doi:10.1080/14756366.2023.2185762)
Supplement: Supplemental Material [file IENZ_A_2185762_SM0422.pdf]

Supporting Information

Potent carbonic anhydrase I, II, IX and XII inhibition activity of novel primary benzenesulfonamides incorporating bis-ureido moieties

Tuba Tekeli[a,b], Suleyman Akocak[c*], Andrea Petreni[d], Nebih Lolak[c], Servet Çete[b], Claudiu T. Supuran[d*]

[a]*Vocational School of Technical Science, Department of Chemistry and Chemical Processing Technologies, Adıyaman University, 02040 Adıyaman, Türkiye*

[b]*Department of Chemistry, Faculty of Science, Gazi University, Ankara, Türkiye*

[c]*Department of Pharmaceutical Chemistry, Faculty of Pharmacy, Adıyaman University, 02040 Adıyaman, Türkiye*

[d]*Università degli Studi di Firenze, NEUROFARBA Dept., Sezione di Scienze Farmaceutiche, Via Ugo Schiff 6, 50019 Sesto Fiorentino (Florence), Italy*

*Corresponding authors: Tel: +90 (416) 223 3800-2836, Fax: +90 (416) 223 3842, E-mail: akocaksuleyman@gmail.com, sakocak@adiyaman.edu.tr, (Suleyman Akocak);
Tel/Fax: +39-055-4573729, E-mail: claudiu.supuran@unifi.it (Claudiu T. Supuran).

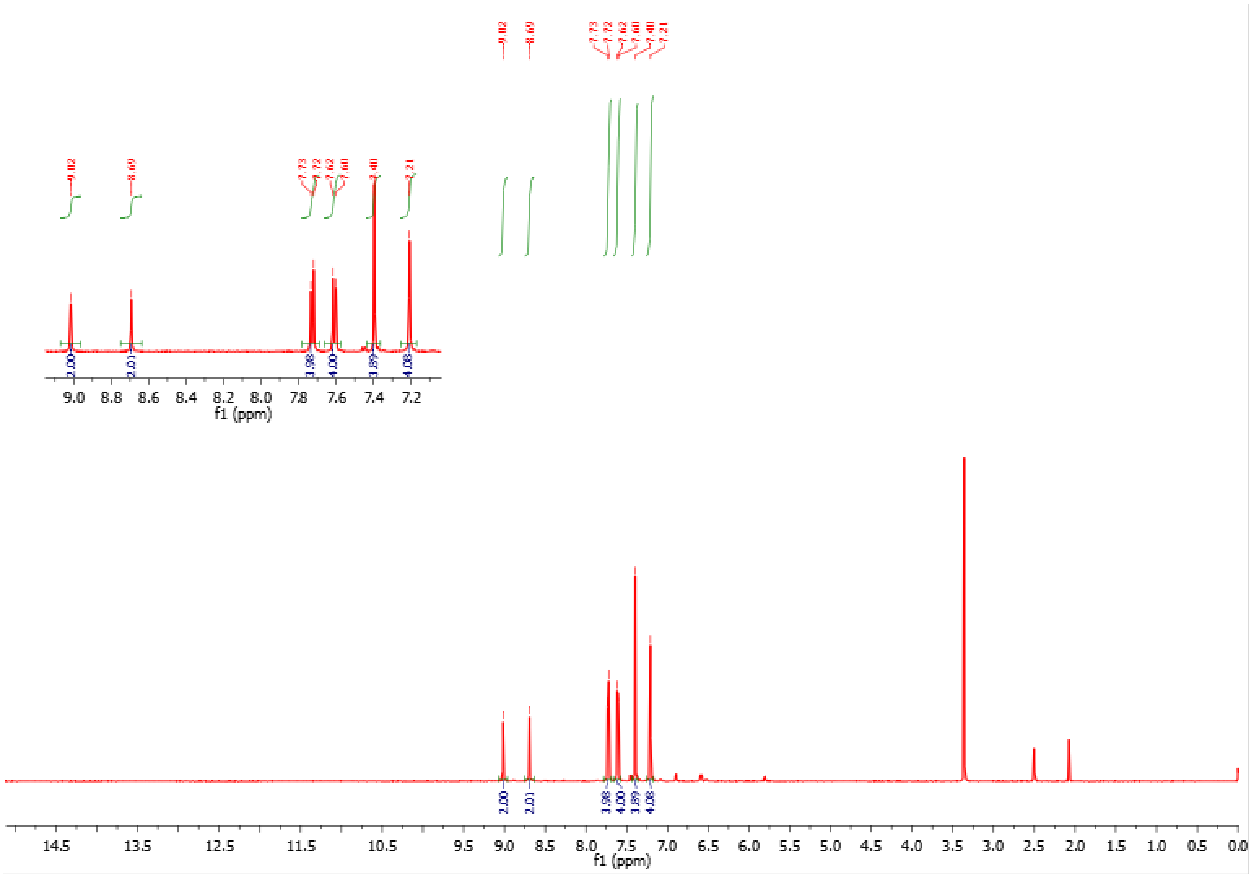

Figure S1: ^{1}H NMR spectrum of compound **8** (**X1Y1**) (500 MHz, in DMSO-d_6).

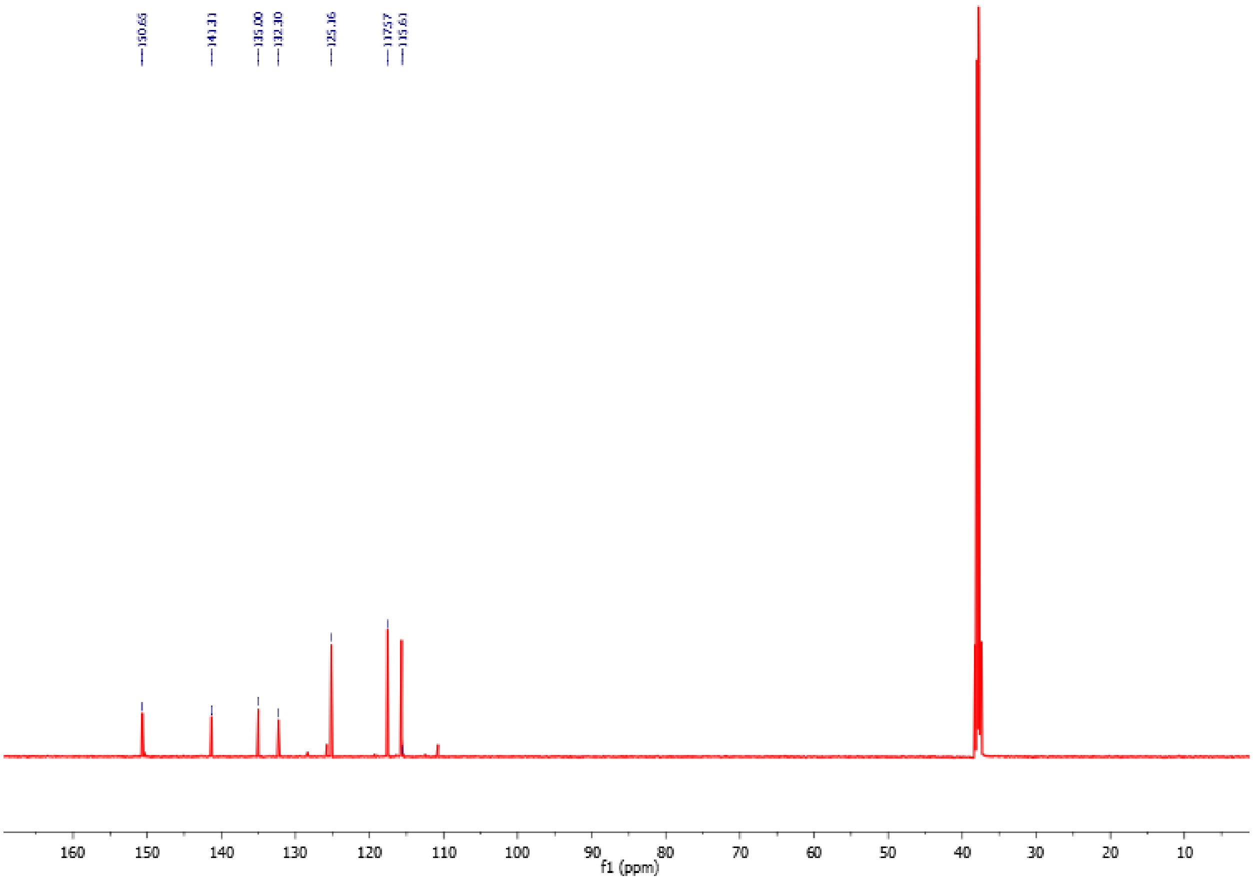

Figure S2: ^{13}C NMR spectrum of compound **8** (**X1Y1**) (125 MHz, in DMSO-d_6).

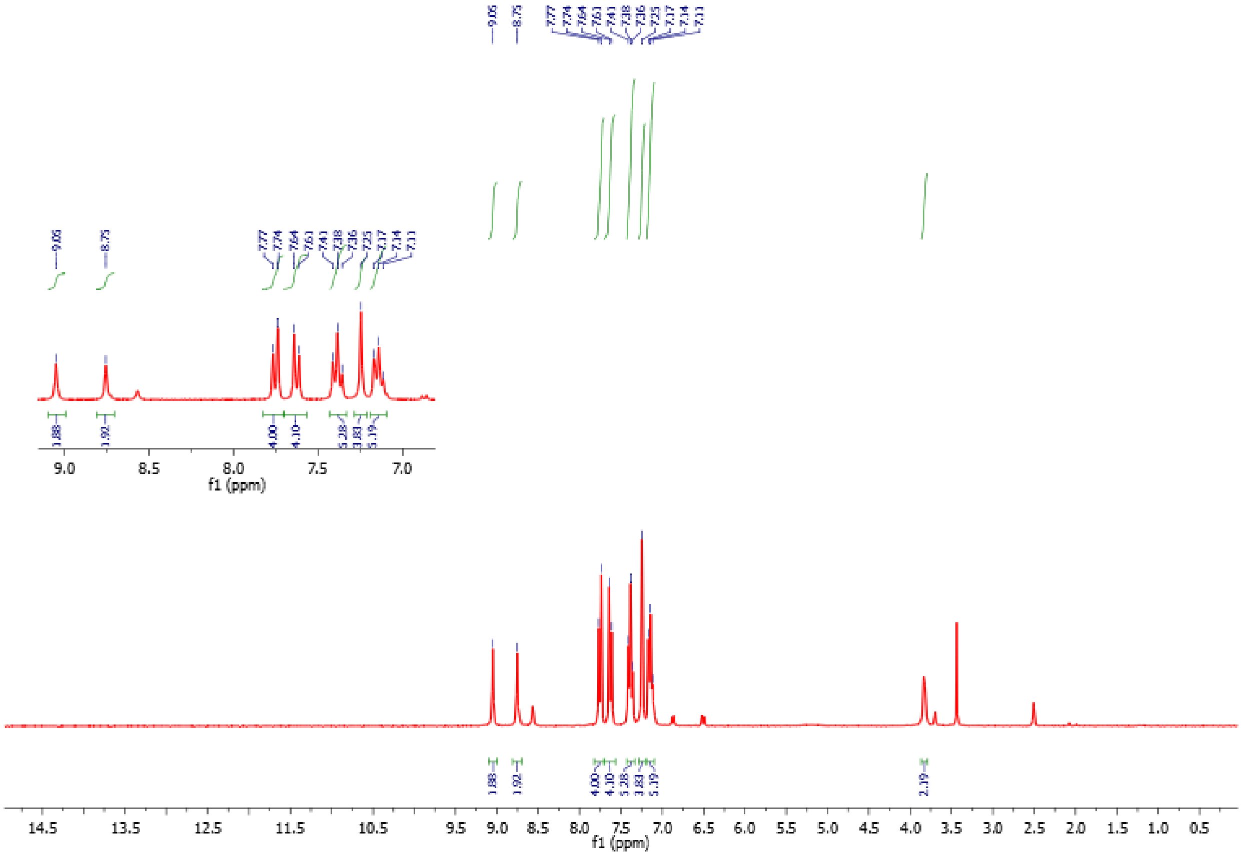

Figure S3: ^{1}H NMR spectrum of compound **9** (**X1Y2**) (500 MHz, in DMSO-d_6).

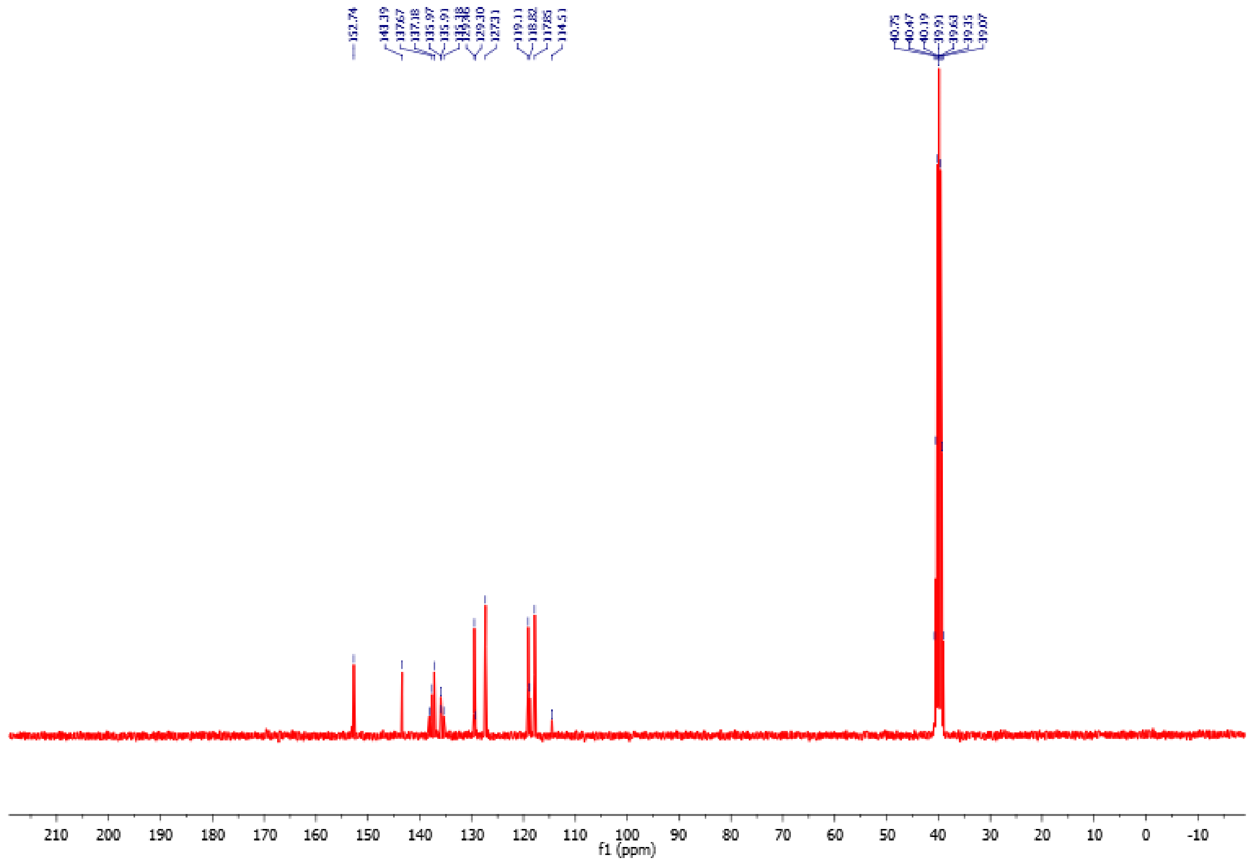

Figure S4: ^{13}C NMR spectrum of compound **9** (**X1Y2**) (125 MHz, in DMSO-d_6).

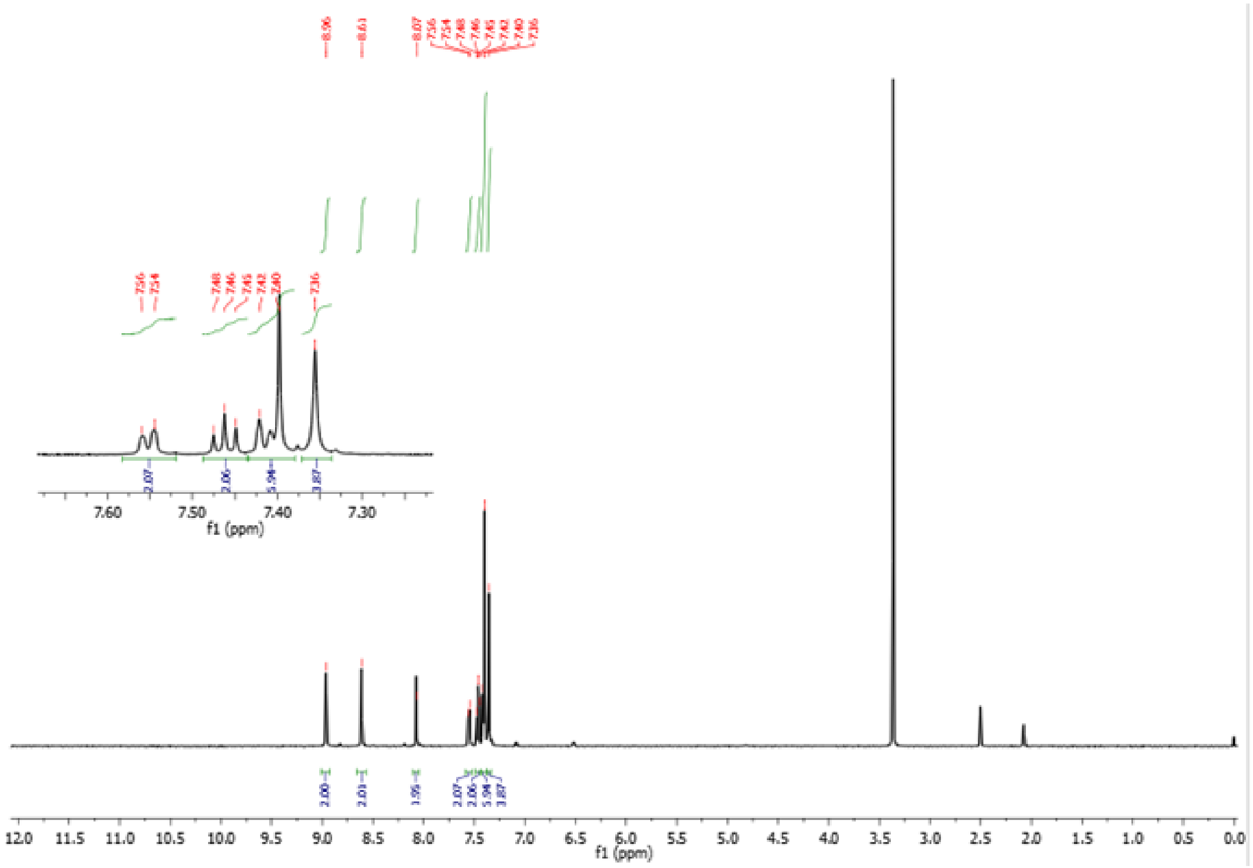

Figure S5: ^{1}H NMR spectrum of compound **12** (**X2Y1**) (500 MHz, in DMSO-d_6).

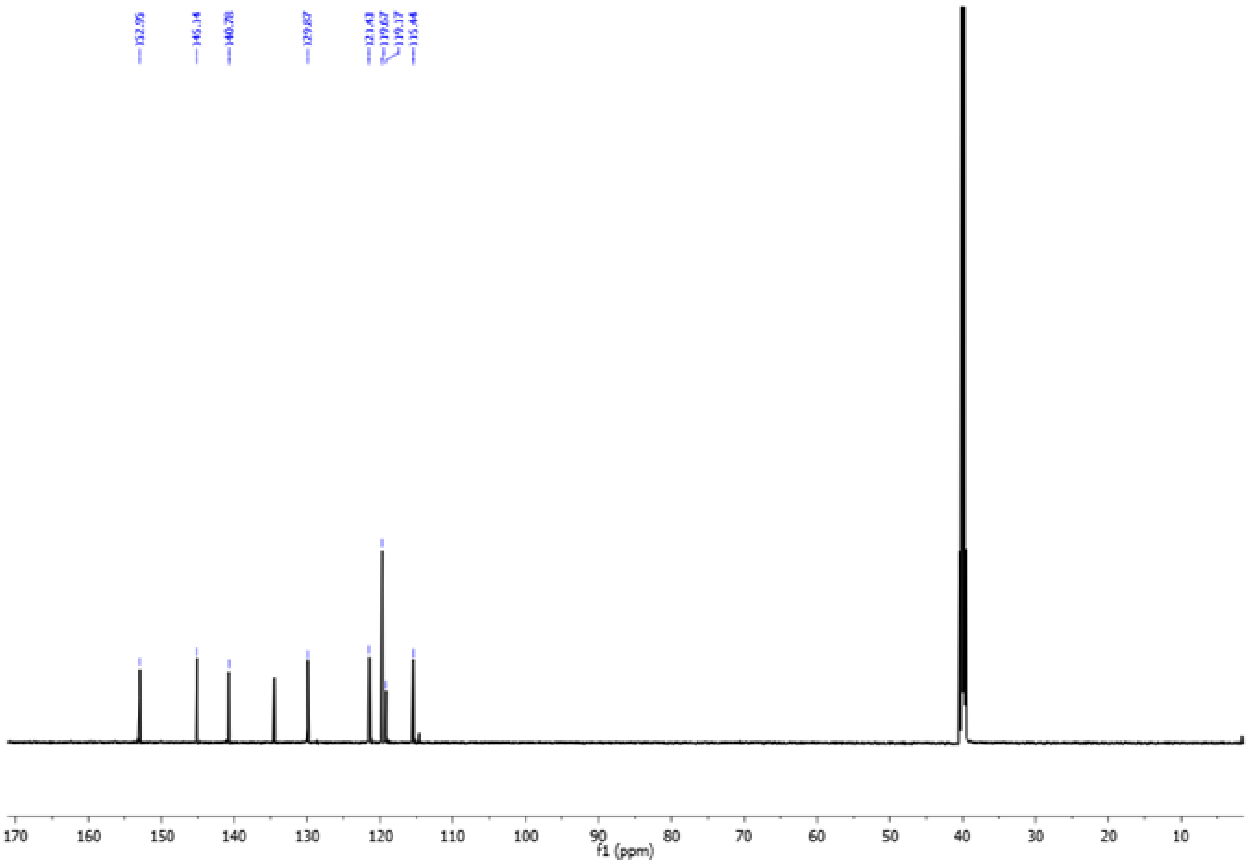

Figure S6: ^{13}C NMR spectrum of compound **12** (**X2Y1**) (125 MHz, in DMSO-d_6).

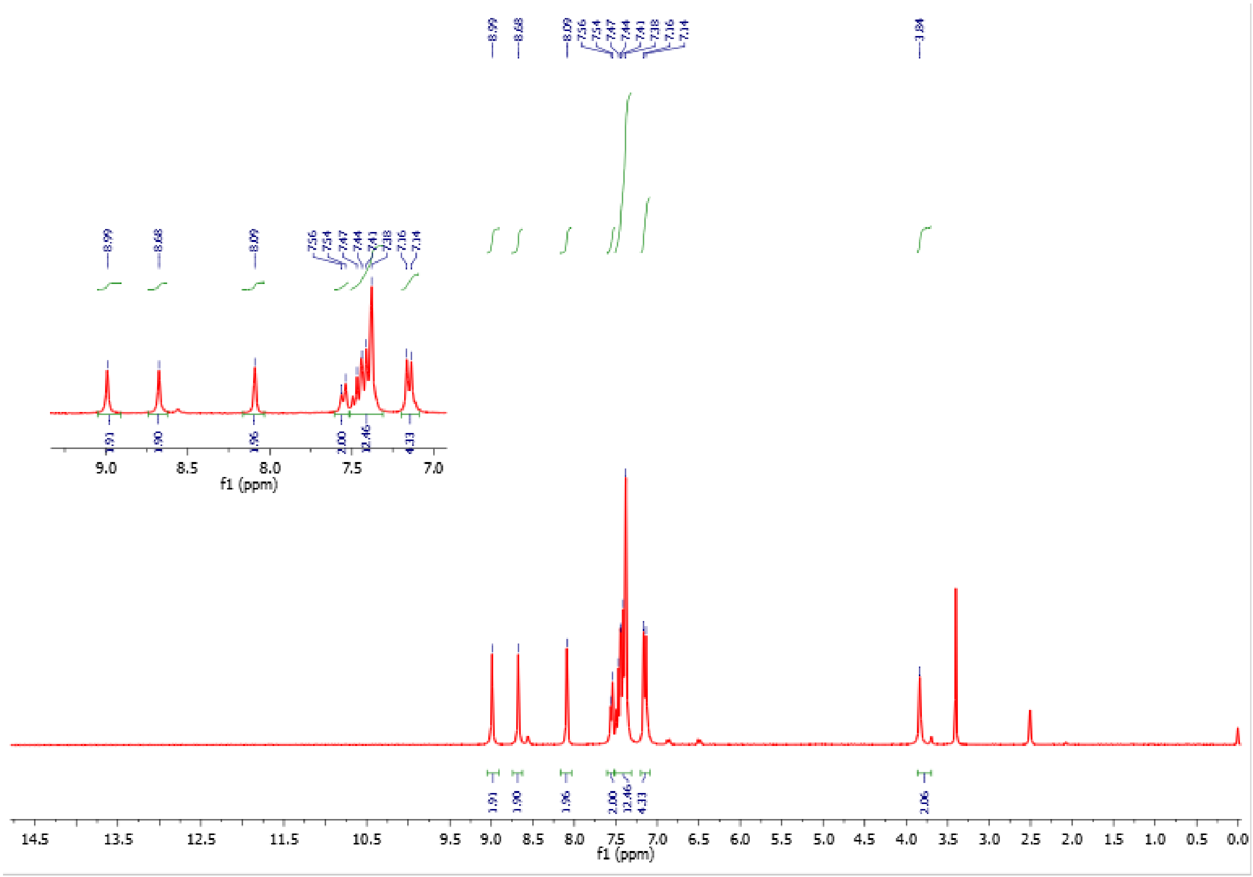

Figure S7: ^{1}H NMR spectrum of compound **13** (**X2Y2**) (500 MHz, in DMSO-d_6).

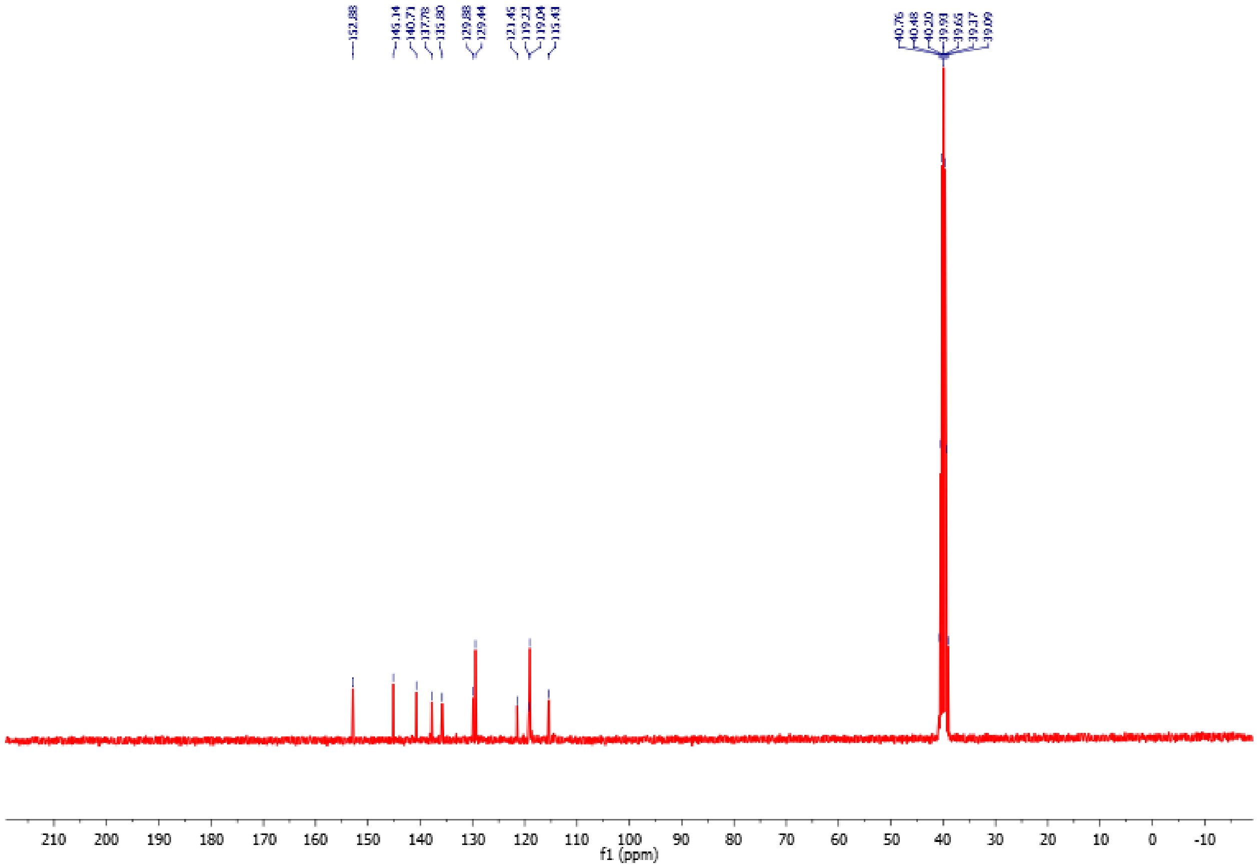

Figure S8: ^{13}C NMR spectrum of compound **13** (**X2Y2**) (125 MHz, in DMSO-d_6).

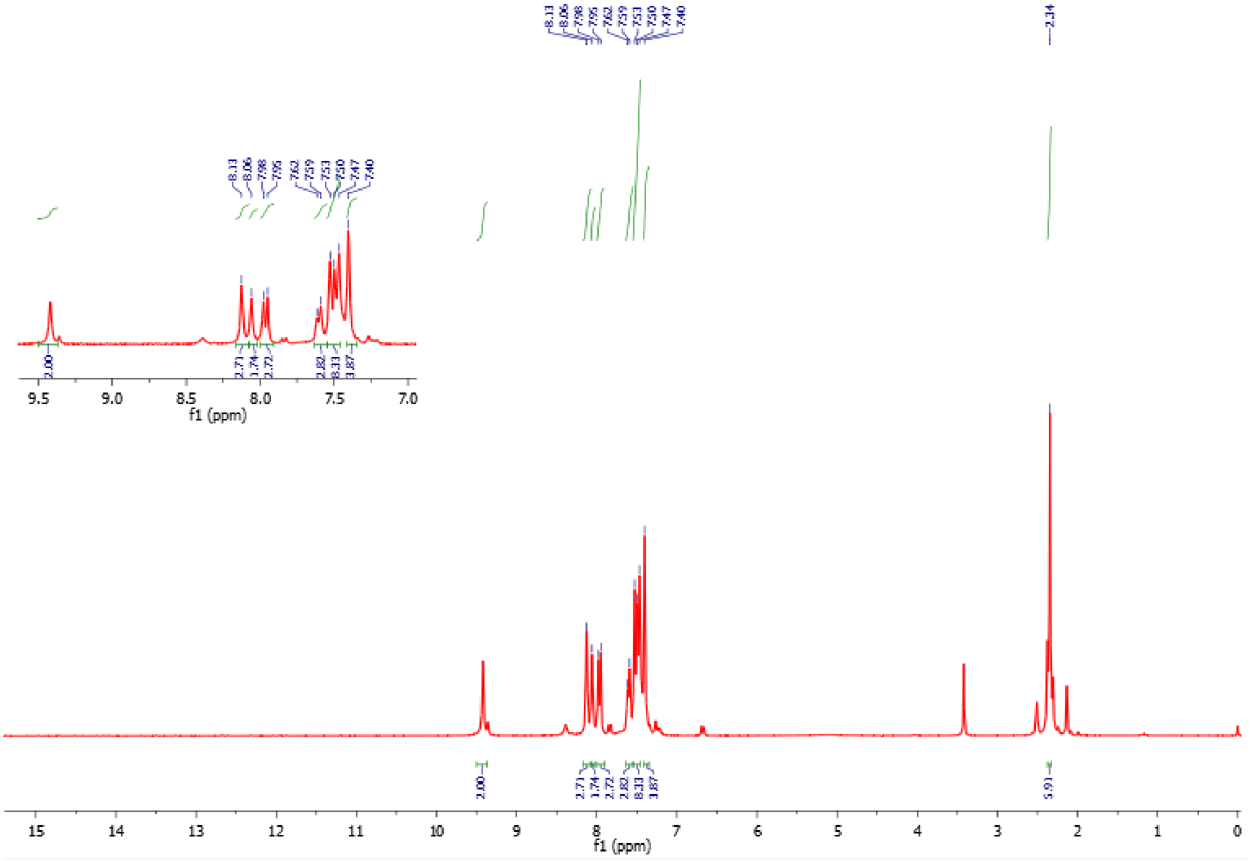

Figure S9: ^{1}H NMR spectrum of compound **14** (**X2Y3**) (500 MHz, in DMSO-d_6).

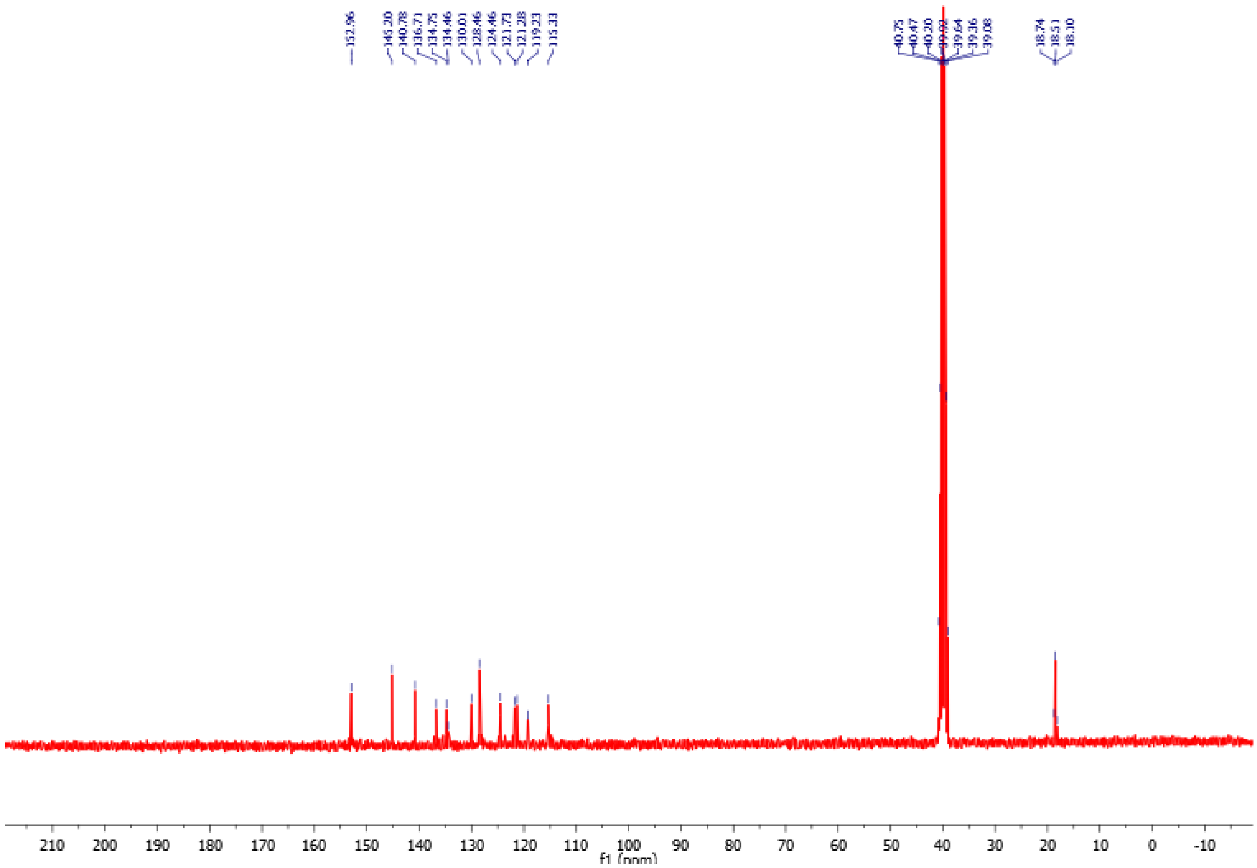

Figure S10: ^{13}C NMR spectrum of compound **14** (**X2Y3**) (125 MHz, in DMSO-d_6).

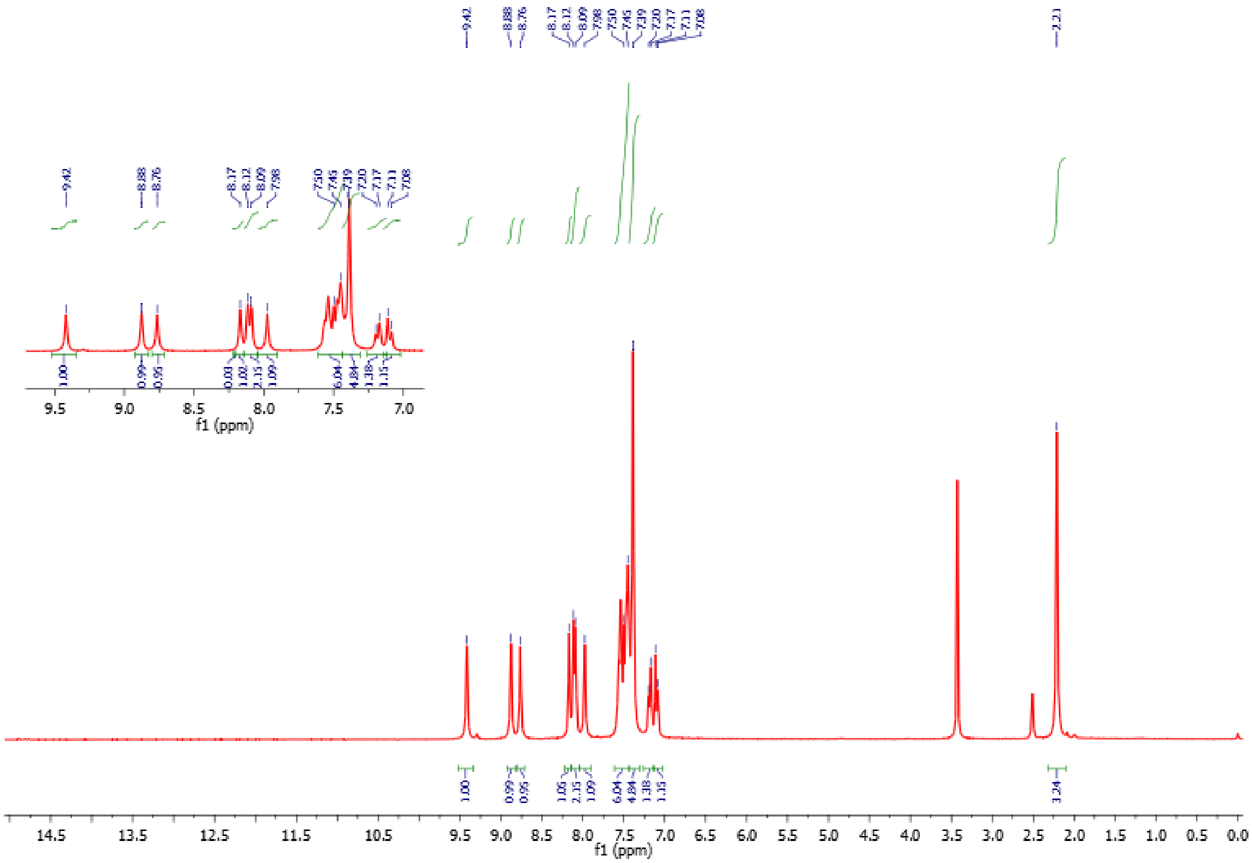

Figure S11: ^{1}H NMR spectrum of compound **15** (**X2Y4**) (500 MHz, in DMSO-d_6).

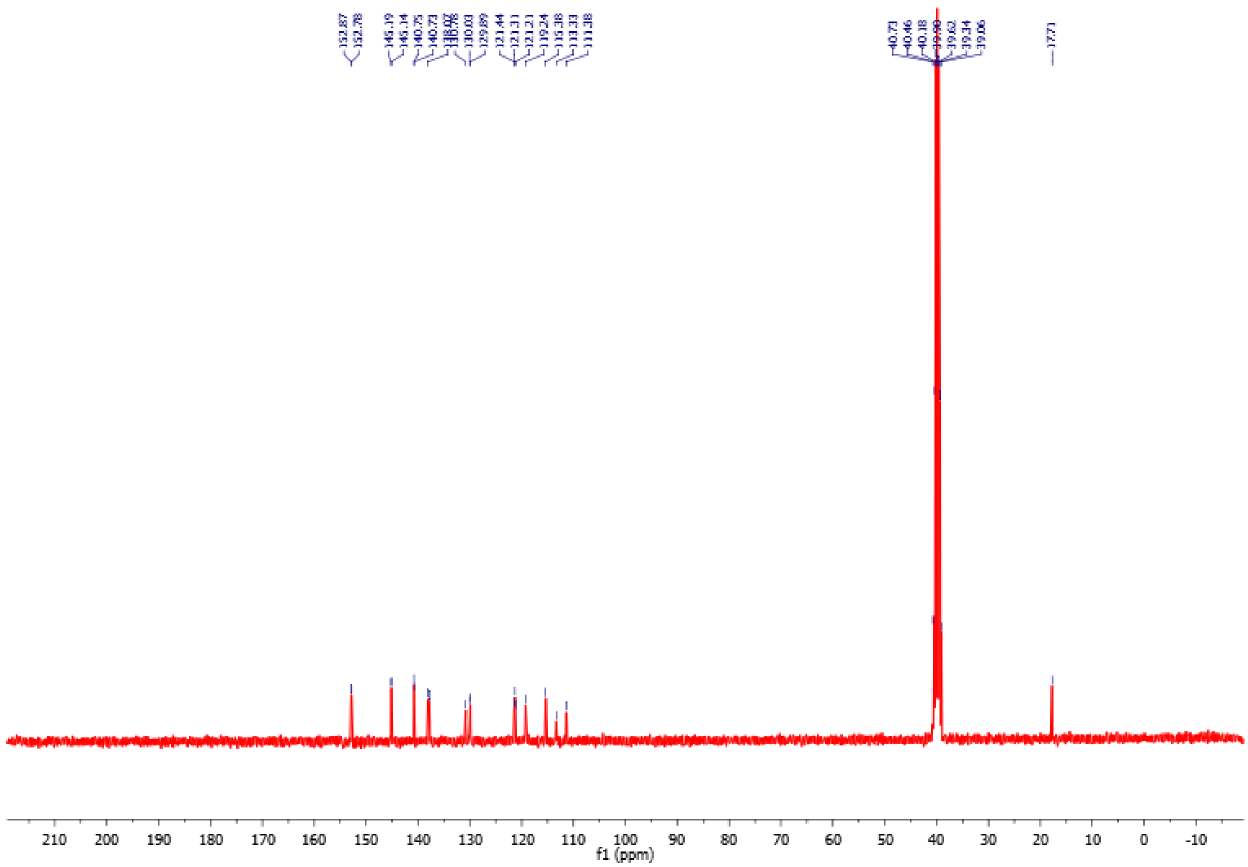

Figure S12: ^{13}C NMR spectrum of compound **15** (**X2Y4**) (125 MHz, in DMSO-d_6).

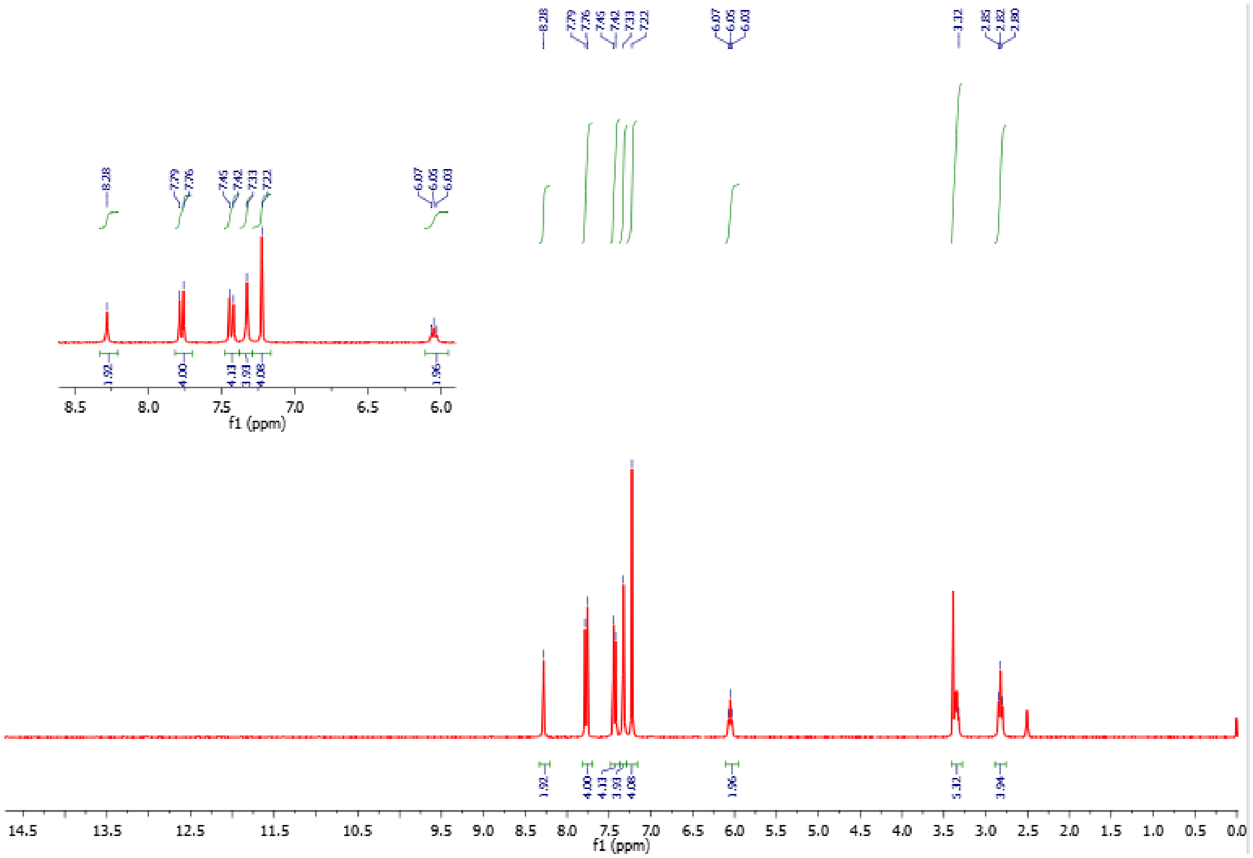

Figure S13: ^{1}H NMR spectrum of compound **16** (**X3Y1**) (500 MHz, in DMSO-d_6).

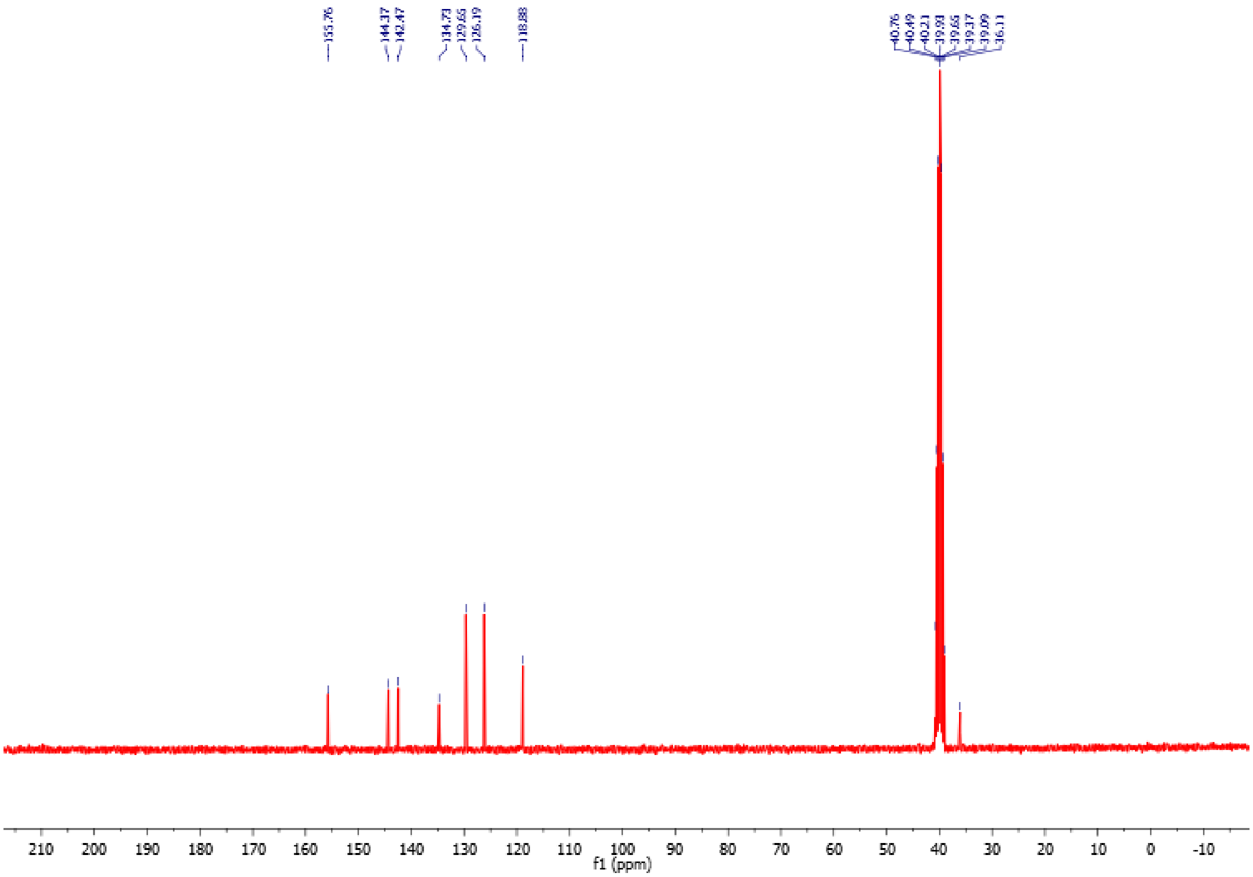

Figure S14: ^{13}C NMR spectrum of compound **16** (**X3Y1**) (125 MHz, in DMSO-d_6).

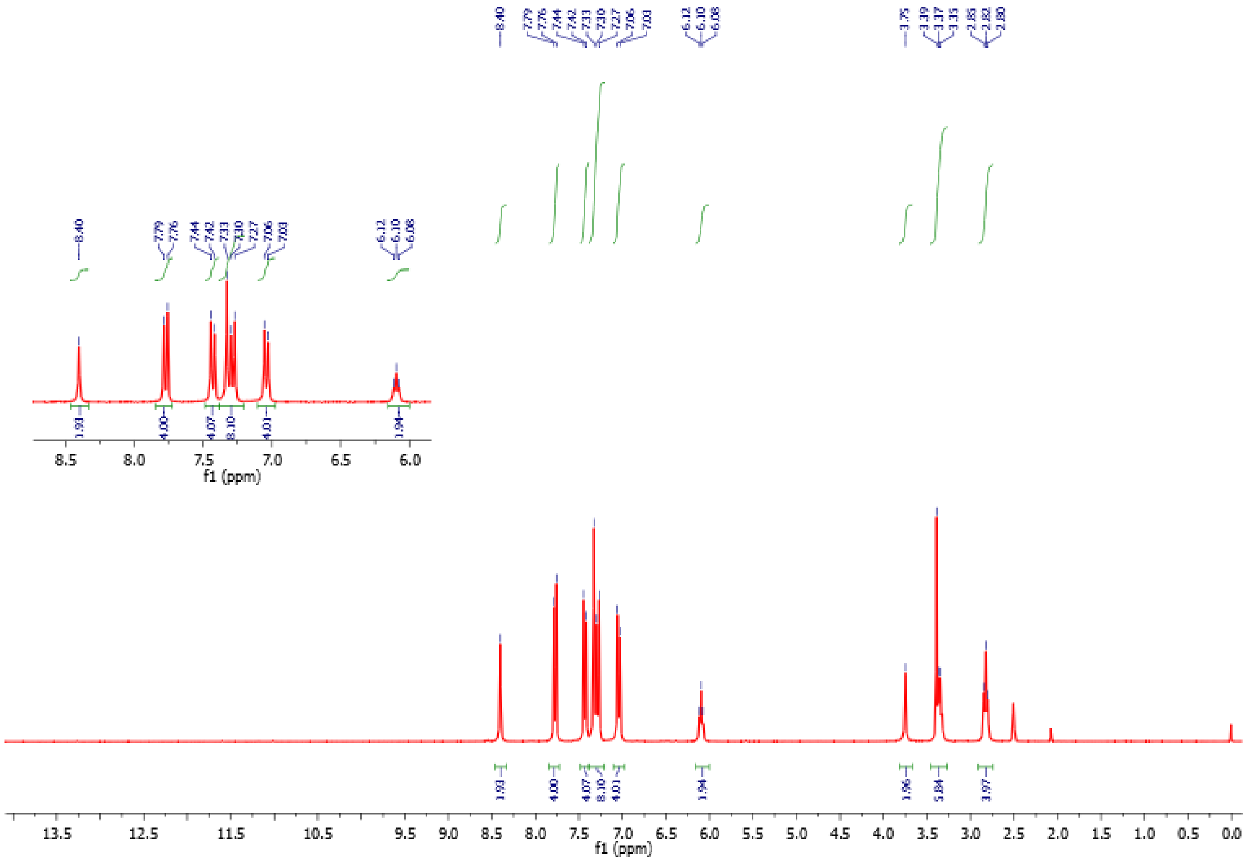

Figure S15: ^{1}H NMR spectrum of compound **17** (**X3Y2**) (500 MHz, in DMSO-d_6).

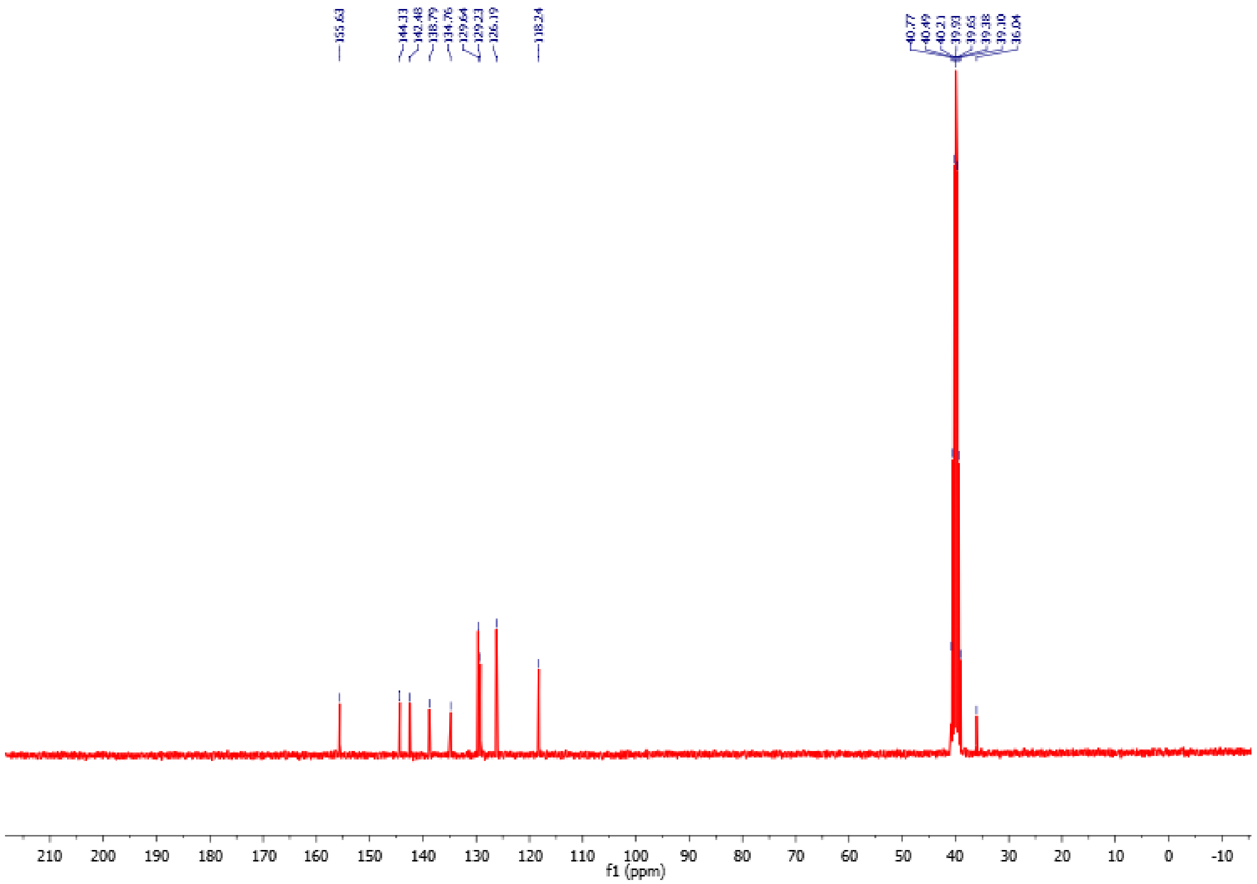

Figure S16: ^{13}C NMR spectrum of compound **17** (**X3Y2**) (125 MHz, in DMSO-d_6).

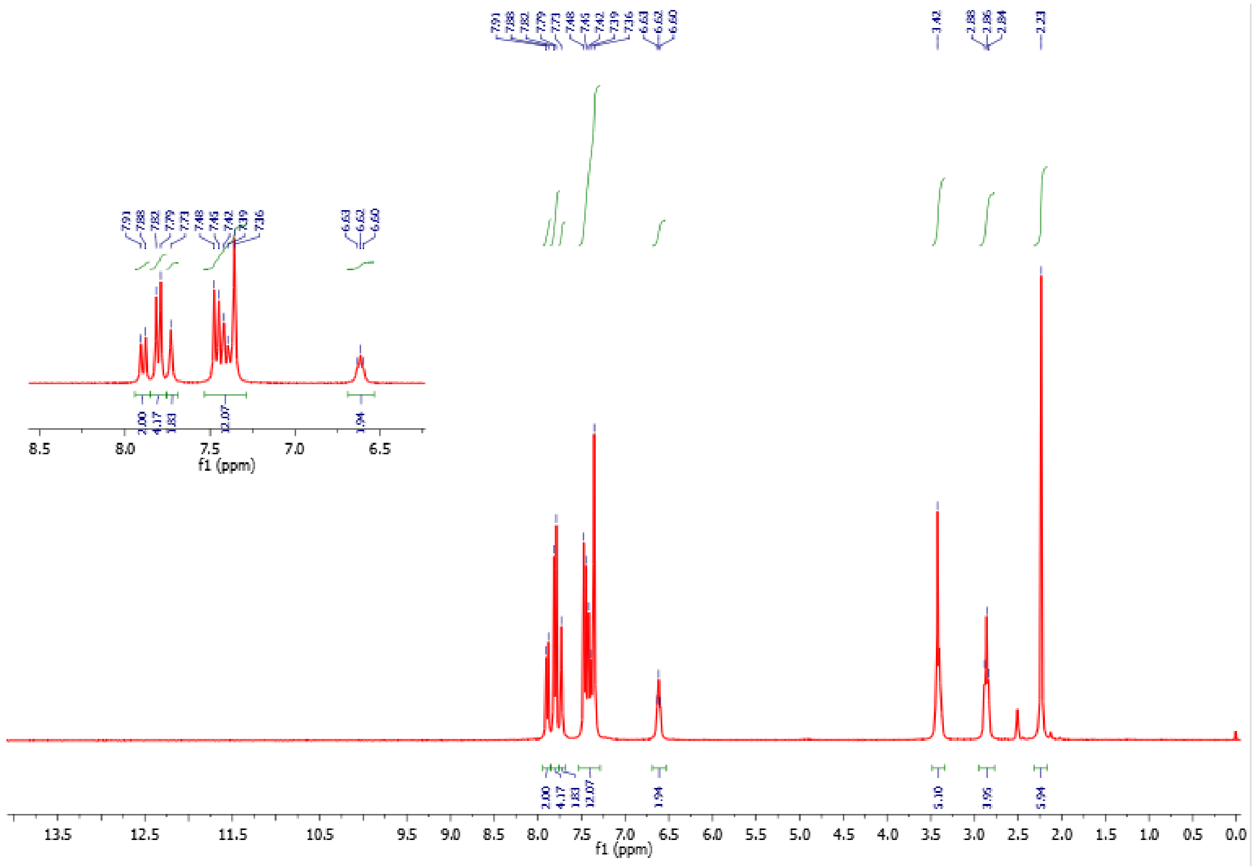

Figure S17: ^{1}H NMR spectrum of compound **18** (**X3Y3**) (500 MHz, in DMSO-d_6).

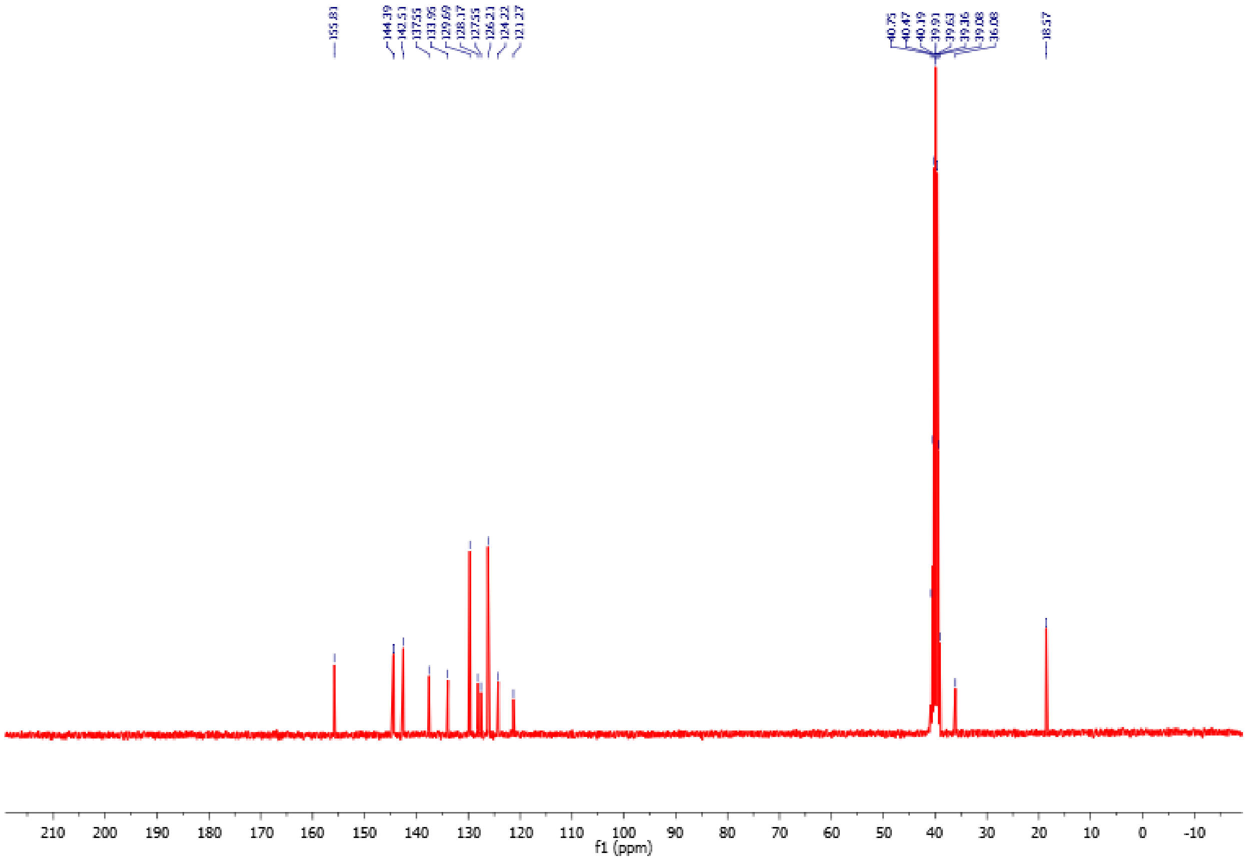

Figure S18: ^{13}C NMR spectrum of compound **18** (**X3Y3**) (125 MHz, in DMSO-d_6).

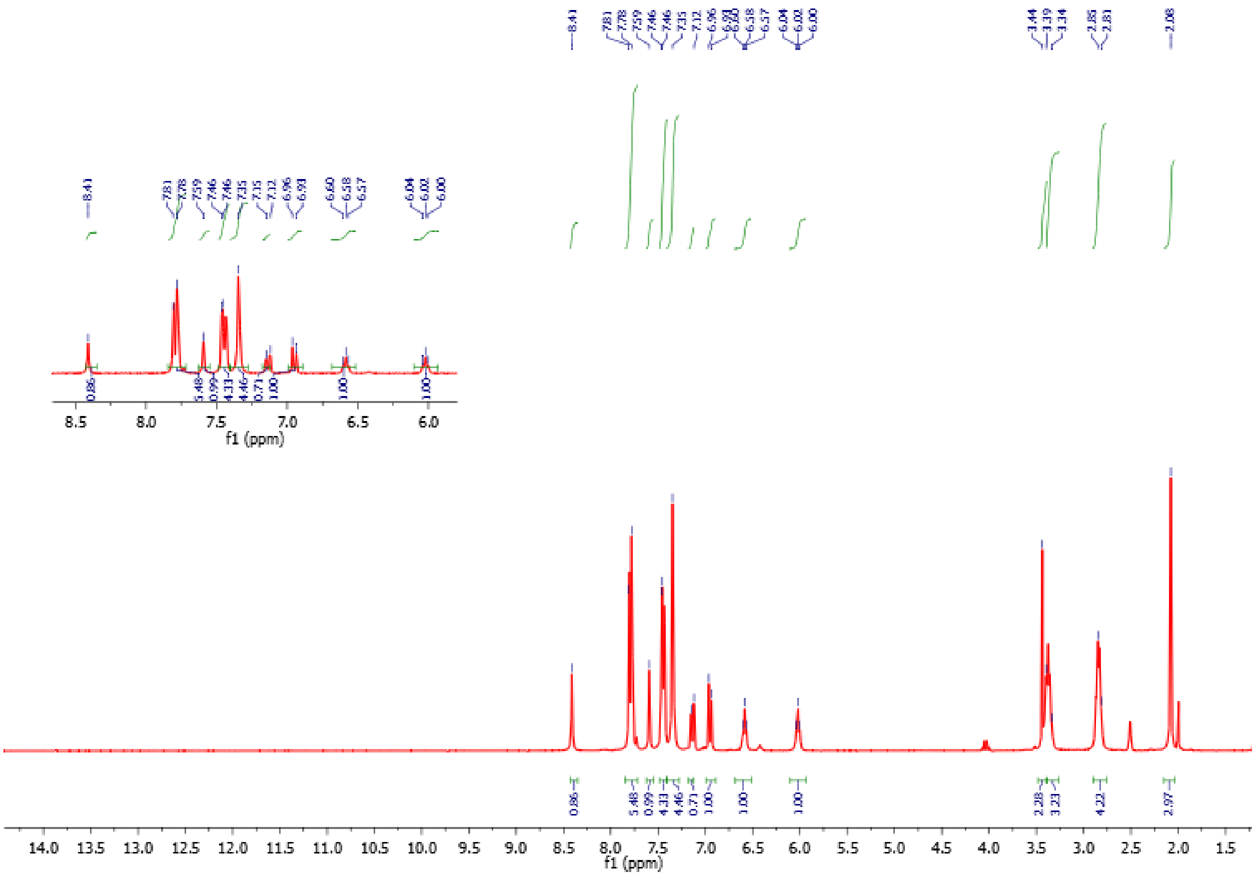

Figure S19: ^{1}H NMR spectrum of compound **19** (**X3Y4**) (500 MHz, in DMSO-d_6).

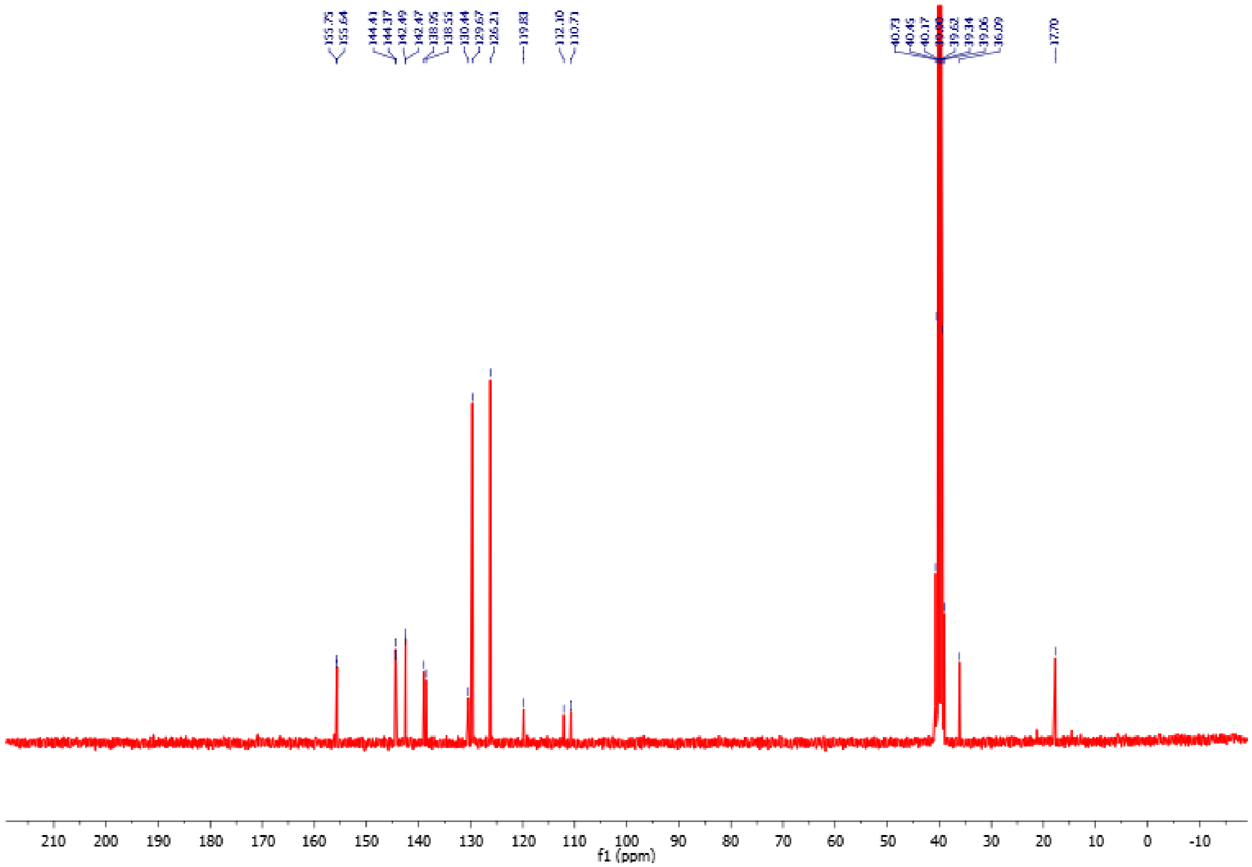

Figure S20: ^{13}C NMR spectrum of compound **19** (**X3Y4**) (125 MHz, in DMSO-d_6).
